# Effect of Strength Training Programs in Middle- and Long-Distance Runners’ Economy at Different Running Speeds: A Systematic Review with Meta-analysis

**DOI:** 10.1007/s40279-023-01978-y

**Published:** 2024-01-02

**Authors:** Cristian Llanos-Lagos, Rodrigo Ramirez-Campillo, Jason Moran, Eduardo Sáez de Villarreal

**Affiliations:** 1 Physical Performance Sports Research Center (PPSRC), Universidad Pablo de Olavide, 41704 Seville, Spain; 2 Exercise and Rehabilitation Sciences Institute, School of Physical Therapy, Faculty of Rehabilitation Sciences, Universidad Andres Bello, 7591538 Santiago, Chile; 3 School of Sport, Rehabilitation and Exercise Sciences, University of Essex, Colchester, Essex CO43SQ UK

## Abstract

**Background:**

Running economy is defined as the energy demand at submaximal running speed, a key determinant of overall running performance. Strength training can improve running economy, although the magnitude of its effect may depend on factors such as the strength training method and the speed at which running economy is assessed.

**Aim:**

To compare the effect of different strength training methods (e.g., high loads, plyometric, combined methods) on the running economy in middle- and long-distance runners, over different running speeds, through a systematic review with meta-analysis.

**Methods:**

A systematic search was conducted across several electronic databases including Web of Science, PubMed, SPORTDiscus, and SCOPUS. Using different keywords and Boolean operators for the search, all articles indexed up to November 2022 were considered for inclusion. In addition, the PICOS criteria were applied: *Population*: middle- and long-distance runners, without restriction on sex or training/competitive level; *Intervention*: application of a strength training method for ≥ 3 weeks (i.e., high loads (≥ 80% of one repetition maximum); submaximal loads [40–79% of one repetition maximum); plyometric; isometric; combined methods (i.e., two or more methods); *Comparator*: control group that performed endurance running training but did not receive strength training or received it with low loads (< 40% of one repetition maximum); *Outcome*: running economy, measured before and after a strength training intervention programme; *Study design*: randomized and non-randomized controlled studies. Certainty of evidence was assessed with the GRADE approach. A three-level random-effects meta-analysis and moderator analysis were performed using R software (version 4.2.1).

**Results:**

The certainty of the evidence was found to be moderate for high load training, submaximal load training, plyometric training and isometric training methods and low for combined methods. The studies included 195 moderately trained, 272 well trained, and 185 highly trained athletes. The strength training programmes were between 6 and 24 weeks’ duration, with one to four sessions executed per week. The high load and combined methods induced small (ES = − 0.266, *p* = 0.039) and moderate (ES = − 0.426, *p* = 0.018) improvements in running economy at speeds from 8.64 to 17.85 km/h and 10.00 to 14.45 km/h, respectively. Plyometric training improved running economy at speeds ≤ 12.00 km/h (small effect, ES = − 0.307, *p* = 0.028, *β*_1_ = 0.470, *p* = 0.017). Compared to control groups, no improvement in running economy (assessed speed: 10.00 to 15.28 and 9.75 to 16.00 km/h, respectively) was noted after either submaximal or isometric strength training (all, *p* > 0.131). The moderator analyses showed that running speed (*β*_1_ = − 0.117, *p* = 0.027) and *V*O_2_max (*β*_1_ = − 0.040, *p* = 0.020) modulated the effect of high load strength training on running economy (i.e., greater improvements at higher speeds and higher *V*O_2_max).

**Conclusions:**

Compared to a control condition, strength training with high loads, plyometric training, and a combination of strength training methods may improve running economy in middle- and long-distance runners. Other methods such as submaximal load training and isometric strength training seem less effective to improve running economy in this population. Of note, the data derived from this systematic review suggest that although both high load training and plyometric training may improve running economy, plyometric training might be effective at lower speeds (i.e., ≤ 12.00 km/h) and high load strength training might be particularly effective in improving running economy (i) in athletes with a high *V*O_2_max, and (ii) at high running speeds.

**Protocol Registration:**

The original protocol was registered (https://osf.io/gyeku) at the Open Science Framework.

**Supplementary Information:**

The online version contains supplementary material available at 10.1007/s40279-023-01978-y.

## Key Points

**Table:** 

Strength training with high loads (≥ 80% of one repetition maximum) can improve running economy and might be particularly effective in athletes running at high speeds (e.g., > 12.00 km/h) and/or possessing a well-developed *V*O_2_max.
Plyometric training could improve running economy at speeds less than 12.00 km/h.
The combination of two or more strength training methods (e.g., high load training, plyometric training) may induce greater running economy improvement, compared to isolated training methods.
These results are based on 31 studies with moderate to low certainty of evidence for the main outcomes, involving a total of 652 middle- and long-distance runners.

## Introduction

Concurrent training involves the execution of both endurance and strength training within the same training cycle [1] and is effective in enhancing the running performance [2, 3] and running economy (RE) [2, 4, 5] of middle- and long-distance runners. RE is defined as the energy demanded at submaximal running speeds [6] and is one of the key determinants of overall running performance along with maximal oxygen uptake (*V*O_2_max), anaerobic threshold and anaerobic capacity [7, 8]. However, a variety of other strength training methods, or combinations thereof, may generate different types of adaptations [2] that could affect changes in RE and, whilst the different protocols used to measure RE may also affect the results of the effect of strength training [2, 9].

RE has been shown to be associated with performance in middle- and long- distance running [10–12]. It has been found that trained runners have better RE than active untrained subjects [13]. Indeed, in athletes with a similar *V*O_2_max, those with better RE have demonstrated better running performance [11] because they can run at a higher relative intensity or maintain a constant intensity for a relatively longer period of time [14]. One of the strategies for improving RE has traditionally been strength training which can generate various neuromuscular adaptations such as improved intra- and inter-muscular coordination, improved muscle–tendon stiffness, and increased recruitment and firing rate of motor units, in addition to morphological changes [15]. However, strength training programmes can be designed in multiple different ways by varying some of the training parameters (e.g., load, number of sets and repetitions, exercise sequence) [16], which could induce different neuromuscular adaptations in middle- and long-distance runners [15]. For example, strength training with high loads [HL, i.e., ≥ 80% 1 repetition maximum (1 RM)], submaximal loads (SL, i.e., 40–79% 1 RM), isometric training (ISO) and plyometric training (PL) can enhance maximal strength development, strength at submaximal loads, isometric force production and stretch-shortening cycle activity, respectively [15, 17]. In addition, the inclusion of different strength training methods (e.g., HL with PL) have been used as a strategy to improve in different areas of the force–velocity relationship (i.e., different loads and speeds of movements) [18].

In addition to the above, the improvement of RE through strength training has exhibited varying effects depending on the running speed that is being assessed. For example, Piacentini et al. [19] found that strength training with HL only significantly improved RE at marathon pace (*p* < 0.05), but not at 1.00 km/h faster or slower than marathon pace. Another study [20] found that HL, PL, and complex training (i.e., HL with PL executed within the same session) improved RE at a speed of 12.00 km/h, whereas HL and complex training improved it at 14.00 km/h, while complex training only elicited improvements at 16.00 km/h. These differences in results could possibly have occurred due to differences in the measurement of RE (e.g., as energy cost or oxygen cost) and the chosen running speed, which could be classified as absolute speed or relative speed (e.g., speed relative to anaerobic threshold or race pace) [21, 22]. Moreover, a U-shaped relationship between energy cost and running speed has been found because at the lowest and highest speeds, there appears to be varying utilisation of the stretch-shortening cycle and activation of skeletal muscle in the muscle–tendon unit [23]. In addition, it has been suggested that RE should be measured at a speed relative to the anaerobic threshold [22] as it ensures the same relative intensity for each participant. Accordingly, several methodological aspects related to running speed should be considered when assessing changes in RE. Although there are several systematic reviews and meta-analyses that have examined the effect of strength training on RE [2, 4, 5, 24], none of these have assessed the effect of different strength training methods on RE and the influence of running speed used in the assessment of RE.

Therefore, this systematic review with meta-analysis aimed to compare the effect of different strength training methods on the RE in middle- and long-distance runners, over different running speeds.

## Methods

This systematic review and meta-analysis was conducted according to the guidelines established by the PRISMA statement [25]. The protocol was registered prior to analysis of the data at the Open Science Framework (https://osf.io/gyeku).

### Information Sources and Search Strategy

The search for articles was carried out in PubMed, Web of Science (all databases), Scopus and SPORTDiscus databases. All articles indexed up to January 2022 were included for the selection. Different terms and Boolean operators were used for the search [Table S1 in the Online Supplementary Material (OSM)]. No limits of study design, date, language, age, or sex were imposed on the search. The search was updated in November 2022, through notifications of new studies found in the search strategy in the different databases. In addition, the reference lists of includable articles, and from reviews, systematic reviews and meta-analyses retrieved from our search were scanned for additional articles of interest.

### Selection Process

All titles and abstracts obtained from the database searches were screened independently by two authors (LL and SV) and those potentially meeting the inclusion criteria (Table 1) were included for full text analysis. In the case of a disagreement between the two authors, a third author (RRC) was consulted.

**Table 1:** Inclusion and exclusion criteria for meta-analysis

Category	Inclusion criteria	Exclusion criteria
Population	Amateur and competitive middle- and long-distance runners (i.e., running distances ≥ 1500 m), aged > 16 years old, without restriction to sex or training/competitive level	Subject with injuries, comorbidities, or non-runners endurance athletes
Intervention	A strength training programme (i.e., HL, SL, PL, ISO, or a combination of them) which was in addition to, or in partial replacement (i.e., matched training load) of endurance running training, lasting ≥ 3 weeks, with ≥ 1 weekly session	The programme includes alternative methods in addition to strength training (e.g., electrical stimulation or body vibration), and/or supplementations (e.g., creatin)
Comparator	Control group that performed endurance running training but did not receive strength training or received it with low loads (< 40% 1 RM or > 20 RM)	Absence of control group
Outcome	Running economy was recorded in at least one speed before and after the strength training intervention	Baseline and/or follow-up data not available
Study design	Randomised and non-randomized controlled studies	Cross-sectional, observational, or case studies

*HL* high load training, *ISO* isometric training, *PL* plyometric training, *RM* repetition maximum, *SL* submaximal training, *1 RM* one repetition maximum

### Eligibility Criteria

Studies were eligible for inclusion according to the participants, intervention, comparator, outcome, and study design (PICOS) criteria (Table 1).

### Data Collection Process

From included studies, an independent reviewer (LL) extracted the data regarding participants characteristics, intervention characteristics, and the main outcomes (means and standard deviations). In those articles where only figure data were available, the validated (*r* = 0.99, *p* < 0.001) [26] software WebPlotDigitizer (version 4.5, Pacifica, California, USA) was used to extract the data. Once recoded, the reviewers (LL, SV and RRC) discussed on disagreements and controversial data.

#### Participants

Only subjects over 16 years of age were considered as pubertal stage can affect physical fitness due to hormonal changes during this period [27]. Subjects were considered to have strength training experience based on the information from each study. According to *V*O_2_max mean values, the performance level was categorized as moderately trained (male ≤ 55 ml/kg/min, female ≤ 45 ml/kg/min), well-trained (male 55–65 ml/kg/min, female 45–55 ml/kg/min) or highly trained (≥ 65 ml/kg/min, ≥ 55 ml/kg/min) [28]. When both sexes were measured without distinction, the ranges established were the average of males and females for the respective performance levels. In the case where *V*O_2_max was not recorded in a study, level performance was based on participants’ level of competition (moderately trained = recreational or local club level; well-trained = collegiate or provincial level; highly trained = national or international level) [9].

#### Strength Training Intervention

Strength training methods (i.e., HL, SL, PL, ISO, or a combination of strength training methods), duration of intervention (i.e., weeks; frequency; total sessions), programming model (i.e., lineal; constant; undulating) and exercise sequence (i.e., traditional training; complex training) were recorded. The strength training programme was considered when it was added to, or partially substituted (i.e., matched the training load) for, endurance running training, with an intervention duration of three weeks or more. This corresponds to the time in which both neural and hypertrophic factors can affect strength gains [29], with at least one session undertaken per week. The strength training methods were classified focusing on load (% 1 RM) and/or training target: HL was defined as a program in which exercise was performed with heavy loads (≥ 80% 1 RM or ≤ 7 RM) that were intended to improve maximal force development (e.g., barbell squat, deadlift, etc.); SL with moderate to low loads (peak power load, 40–79% 1 RM or 8–20 RM) with the aim of improving strength development at SL; PL using light-load exercises (< 40% 1 RM) with the aim of improving the stretch-shortening cycle and muscle–tendon stiffness (e.g., drop jumps) and; ISO using isometric contraction exercises (e.g., isometric mid-thigh pull). Those groups that performed strength training with low loads (< 40% 1 RM or > 20 RM) were considered as a control group. The duration of the intervention was counted as total number of weeks, sessions per week and total sessions. The exercise sequence within the session was classified into two, comprising of traditional sequences where exercises are executed with light loads followed by heavy loads and complex sequences where exercises are executed with heavy loads followed by light loads [18]. In the case that the different strength training methods were performed in separate sessions or in different periods, these were considered as traditional sequences.

#### Outcome Measurements

RE was recorded as the energy demand at submaximal running speed. Of note, *energy* among studies was reported in different units of measurement (i.e., calorie; oxygen). When both units were reported in a study, the unit “calorie” was selected because it considers differences in substrate use and is more sensitive to changes in speed [21]. In addition, when the respiratory exchange ratio (RER) is less than one, oxidative metabolism is the main metabolic pathway; thus, when the RER is greater than one, and blood lactate values were presented, the energy value was corrected with the energy values of blood lactate [30]. RE values were collected for all speeds assessed in the studies and for methodological purposes, different variables were generated from this data. In RE at absolute speed (km/h), the absolute speed was collected and, when data were presented in relation to a physiological value [e.g., anaerobic threshold or velocity at *V*O_2_max (v*V*O_2_max)] or a race pace (e.g., 3000 m race pace), we calculated the speed from the average baseline values of both groups (i.e., experimental and control groups). Categorical speeds (≤ 12.00 km/h or > 12.00 km/h) were generated from the mean value of all speeds reported in the included studies. On the other hand, when RE is assessed as absolute speed, the difference in substrate energy relative to anaerobic threshold and *V*O_2_max is not considered [21]. Therefore, two categories were generated relative to anaerobic threshold (i.e., second lactate threshold, onset of blood lactate accumulation, maximal lactate steady state, lactate turn point, second ventilatory threshold or critical speed) and *V*O_2_max. For RE relative to the anaerobic threshold, speed values were classified: speeds > anaerobic threshold and speeds ≤ anaerobic threshold. If speed values were given as an absolute value and anaerobic threshold values were reported, then the average baseline values of anaerobic threshold of both groups was used to determine the corresponding category. For RE relative to *V*O_2_max, the speed was presented as percentage of v*V*O_2_max or *V*O_2_max (%*V*O_2_max), but when the speed values were presented as an absolute value and v*V*O_2_max was provided, we then calculated the mean baseline v*V*O_2_max of both groups and calculated the percentage corresponding to v*V*O_2_max. In addition, we created a category of the U-shaped RE–speed relationship (< 11.50 km/h, 11.50–14.50 km/h or > 14.50 km/h) [23]. Where the study reported multiple time points (i.e., more than two data points), the first record and the last record immediately after the intervention were recorded.

### Risk of Bias, Publication Bias and Certainty Assessment

The PEDro (Physiotherapy Evidence Database) scale was used to assess the risk of bias of the studies [31, 32]. Of note, in the context of this systematic review, items five to seven from the PEDro scale were removed from the scale because subjects, assessors and researchers are rarely blinded in supervised exercise interventions [31, 33]. Following previous criteria [33], the studies were categorized as follows: ≥ 6 points = “low risk”, 4–5 points = “moderate risk”, and ≤ 3 points = “high risk”. A funnel plot was performed to assess the publication bias of the studies examining each method of strength training. It was considered to have publication bias if an asymmetry was observed in the funnel plot. The funnel plots were built following the R code provided by Fernández-Castilla et al. [34].

The GRADE (Grading of Recommendations Assessment, Development and Evaluation) approach was conducted for rate the certainty of evidence of this systematic review [35–37]. For each of the analyses, we started with a high level of certainty of evidence, which decreases according to the following criteria: risk of bias, downgraded by one level if the median PEDro scale score was moderate risk (< 6 points), or by two levels if it was high risk (< 4 points); inconsistency, downgraded by one level if the *Q*-test for heterogeneity was significant (i.e., *p* < 0.05); indirectness, it was considered low risk because the PICO criteria were assured by default; imprecision, downgraded by one level if the number of participants in the control group with the strength training group was < 800 or if the confidence interval was crossed by a small effect size (i.e., ES = − 0.15 to 0.15); publication bias, downgraded by one level if an asymmetry was observed in the funnel plot.

### Effect Measures

The between-group (i.e., control-experimental) standardized mean difference was calculated as previously recommended [38] and expressed as Hedges’ *g* effect size (ES) [39], which helps to cope with small sample sizes [40], common to sport science literature [41]. When only the mean and standard error (SE) were presented, the standard deviation (SD) was calculated from the SE as follows:

$${\text{SD}}={\text{SE}}\times \sqrt{N}$$

where *N* is the sample size. Thresholds for the magnitude of ESs were set as 0.15, 0.45 and 0.80 for a small, moderate, and large effect, respectively [42].

### Statistical Analyses

A meta-analysis was performed for each strength training method (i.e., HL, SL, PL, ISO, or combined methods) and its effect on RE when at least three studies provided an outcome measure [43]. If a study had two or more comparison groups in the same analysis, the sample size of the control group was divided by the number of intervention groups [44]. In several studies, different speeds were selected to assess RE. In these cases, the procedure is usually to select a representative ES, to synthesise separately the ES for each outcome across all studies, or to average the sizes of the dependent effects within the study [45]. However, in the first two approaches the sample size is reduced decreasing the statistical power [45], while in the third approach it may negatively impact the validity of the results due to overestimation of standard errors [46]. In addition, these procedures cannot assess the potential differences between ES within studies [47, 48]. Therefore, we used a three-level meta-analysis model [47, 48], which is an extension of the random effect meta-analysis model [40], that considers sampling (level 1), within study (level 2) and between study (level 3) variation.

Due to multiple sources of variation between studies (e.g., training and participant characteristics), a randomized effect model with restricted maximum likelihood estimation method was conducted for estimating the parameters model (${\tau }^{2}$) recommended over the traditional DerSimonian and Laird method for continuous data [49]. We based the test statistic and CI in *t*-distribution with Knapp and Husting adjustment [50].

For each of the strength training methods, the heterogeneity of all ES in the data set was analysed using the test for heterogeneity (*Q*-test) [51]. Additionally, the one-side log-likelihood-ratio test (LRT) was performed to determine whether the within-study variance (LRT_level2_) and between-study variance (LRT_level3_) are significant [52]. Outliers were defined as ES in which the upper limit of the 95% confidence interval (95% CI) was lower than the lower limit of the pooled effect confidence interval or the lower limit of the 95% CI higher than the upper limit of the pooled effect confidence interval [53]. A sensitivity analysis was then performed with and without the outlier ES to assess their impact on the analysis [53] (i.e., *p* value from *Q*-test).

The omnibus test (*Q*_*M*_-test) was used to perform a meta-regression (for continuous data), or subgroup analysis (for categorical data), if at least eight studies were pooled. For all analysis alpha was set as 0.05. The three-level meta-analysis were conducted in R (version 4.2.1) with the *metafor* package [51], following the syntax by Assink and Wibbelink [52]. The forest plot for the three-level meta-analysis was performed using the R code provided by Fernández-Castilla et al. [34], and meta-regression and subgroup analysis plots were built with GraphPad Prism 9 (version 9.2.0).

## Results

### Study Selection

**Fig. 1:**
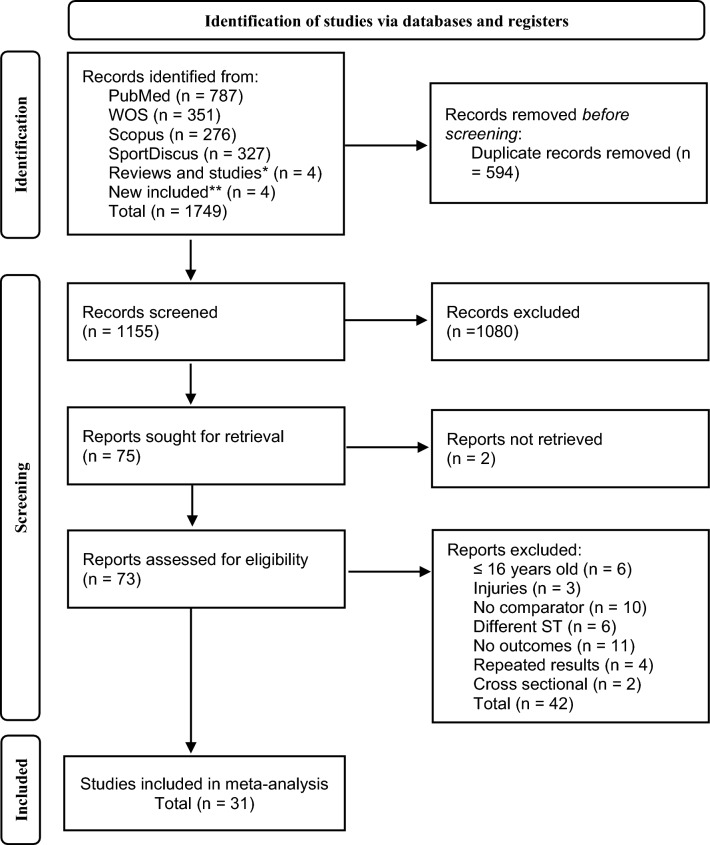
Flow diagram of the study selection process. *Studies found from notifications of new studies found in the search strategy in the different databases. **Studies found in the reference lists of articles, reviews, systematic reviews, and meta-analyses retrieved from our search strategy

A total of 1749 records were identified through the search strategy (Fig. 1). Once duplicate records, records not retrieved and articles excluded after review of titles and/or abstracts were excluded, 73 studies were assessed for eligibility. After reading the full text of each document, 42 studies were excluded due to: participants being under 16 years old [54–59] being injured before intervention [60–62]; studies having no comparator outcomes [63–72]; the strength training method was considered ineligible for inclusion (e.g., core, isokinetic eccentric training and body weight training) [73–78]; RE was not measured [79–89]; outcome results were repeated [90–93] or study was cross-sectional by design [94, 95]. Therefore, 31 studies were included in the final meta-analyses.

### Study Characteristics

A summary of participants’ characteristics and analysis of the studies included in the meta-analysis are presented in Tables 2 and 3, respectively. Thirty-one studies were included in at least one analysis: 11 studies implemented HL, providing 19 ESs [19, 20, 96–104]. Of note, the greater number of ES values (i.e., 19) compared to the number of studies (i.e., 11) indicates that some studies assessed RE at two or more running speeds. Eleven studies implemented PL interventions, providing 28 ESs in total [12, 105–114]. Three studies implemented SL interventions, providing seven ESs [19, 114, 115]. Three studies implemented ISO interventions, providing 7 ESs [112, 116, 117], and nine studies implemented combined methods, providing 20 ESs [8, 20, 102, 115, 118, 119]. The studies included 652 participants [472 males (204 control and 268 treatments) and 180 females (79 control and 101 treatments)], aged between 17 and 45 years old, mean body mass and height of 68.5 kg and 174.3 cm, respectively. Participants were moderately trained (*n* = 195), well trained (*n* = 272), and highly trained (*n* = 185). The strength training programmes were between 6 and 24 weeks’ duration, with one to four sessions per week.

**Table 2:** Participants and strength training intervention characteristics of included studies

Study	G	Subject characteristics							Strength training intervention							
		*n*	Age	BM	H	*V*O_2_max	PLv	ST Exp	D	Fq	TS	Exercises	Load	Set × repetition	Rest	Exercise sequence
Ache-Dias et al. [110]	PL	9 (M = 4; F = 5)	24	63	160	50	MT	NR	8	2	16	PL: Continuous jump	BW	4–6 × 30 s	5 min	Traditional
	C	9 (M = 4; F = 5)	31	66	170	49	MT	NR								
Albracht and Arampatzis [116]	ISO	13 (M)	27	76	180	NR	MT	NR	14	4	56	ISO: Isometric ankle plantarflexion	90% MVC	5 × 4 × 3 s	3 s	Traditional
	C	13 (M)	25	75	178	NR	MT	NR								
Berryman et al.[114]	SL	12 (M)	31	76	176	58	WT	No	8	1	8	SL: Half squat	Power peak load	3–6 × 8	3 min	Traditional
	PL	11 (M)	29	75	178	58	WT	No	8	1	8	PL: Drop jump (20–60 cm)	BW	3–6 × 8	3 min	Traditional
	C	5 (M)	29	76	180	56	WT	No								
Blagrove et al. [9]	SL + PL	9 (M = 4; F = 5)	17	58	170	59	HT	No	10	2	20	PL: Box jump, a-skip, hurdle jump and land, single leg box jump, high-knees, depth jump SL: Back squat, Romanian deadlift, single leg press, calf raises, rack pull, deadlift, step-up	PL: BW. SL: Moderate load	PL: 3–4 × 6–8. SL: 2–3 × 6–12	PL: 1.5 min. SL: 3 min	Traditional
	C	9 (M = 4; F = 5)	18	59	172	62	HT	No								
Damasceno et al. [101]	HL	9 (M)	34	68	174	54	MT	NR	8	2	16	HL: Half squat, leg press, plantar flexion, knee extension	2wk: 75–80% 1RM; 2wk: 80–85%1RM; 2wk: 85–90% 1RM; 2wk: 87–93% 1RM	2–3 × 3–10	3 min	Traditional
	C	9 (M)	33	71	174	56	WT	NR								
do Carmo et al. [108]	PL	15 (M)	33	70	172	55	WT	No	8	2	16	PL: Squat jump, split scissor jump, double leg bound, alternate leg bound, single leg forward hop, depth jump, double leg hurdle jump, single leg hurdle hop	BW	2–5 × 6		
	C	13 (M)	33	70	172	57	WT	No								Traditional
Ferrauti et al. [97]	HL	11 (M = 9; F = 2)	40	77	NR	52	WT	No	8	2	16	HL: Leg press, knee extension, knee flexion, hip extension, ankle extension	87–93% 1RM	4 × 3–5	3 min	Traditional
	C	11 (M = 7; F = 4)	40	70	NR	51	WT	No								
Fletcher et al. [117]	ISO	6 (M)	22	68	180	67	HT	No	8	3	24	ISO: Isometric ankle plantarflexion	80% MVC	3 × 4 × 20 s	1 min	Traditional
	C	6 (M)	26	64	176	68	HT	No								
Johnston et al. [136]	HL + SL	6 (F)	30	57	163	51	WT	No	10	3	30	SL: Bent-leg, heel raise, straight-leg heel raise HL: Knee flexion, knee extension, squat, hammer curl, lunge	SL: 60–67% 1RM. HL: 80–85% 1RM	SL: 2 × 12–20. HL: 3 × 6–8	2 min	Traditional
	C	6 (F)	30	52	164	52	WT	No								
Kelly et al. [100]	HL	7 (F)	21	66	162	40	MT	No	10	3	30	HL: Squat, calf raises, hip extension, hip flexion, hamstring curl	1wk: 60–70% 1RM; 1wk: 70–80% 1RM; 8wk: ≥ 85% 1RM	3 × 5	3–4 min	Traditional
	C	9 (F)	20	60	166	40	MT	No								
Li et al. [20]	HL + PL	10 (M)	20	58	178	66	HT	NR	8	3	24	HL: Back squat, Bulgarian squat, Romanian deadlifts PL: Double leg hurdle hop, single leg hop, drop jump	HL: 80–85% 1RM. PL: BW	HL: 3 × 5 PL: 3 × 6	4 min	Complex
	HL	9 (M)	21	58	175	66	HT	NR	8	3	24	HL: Back squat, Bulgarian squat, Romanian deadlift	80–85% 1RM	5 × 5	3 min	Traditional
	C	9 (M)	21	61	179	66	HT	NR	8	3	24	LL: Back squat, Bulgarian squat, Romanian deadlift	40% 1RM	5 × 20–30	1 min	Traditional
Luckin-Baldwin et al. [135]	SL + HL	14 (M = 10; F = 4)	39	76	177	55	WT	No	24	2	48	SL + HL: Half squat, glute hamstring raises, hip thrusts, single leg press, single leg seated calf raise, hip flexion, hip abduction	12wk: ≥ 75% 1RM; 12wk: ≥ 85% 1RM	1–5 × 1–12		Traditional
	C	11 (M = 9; F = 2)	36	76	176	53	WT	No								
Lum et al. [105]	PL	7 (M)	29	66	171	54	MT	NR	6	2	12	PL: Alternate leg bounding, double leg and single leg hurdle hop, depth jump	BW	3–4 × 5–40	3 min	Traditional
	C	7 (M)	29	66	171	54	MT	NR								
Lum et al. [112]	PL	9 (M = 6; F = 3)	38	65	170	51	WT	Yes	6	2	12	PL: Depth jump, single leg bounding, split jump	BW	2–4 × 4–5	3 min	Traditional
	ISO	9 (M = 6; F = 3)	37	63	173	52	WT	Yes	6	2	12	ISO: Isometric ankle plantarflexion	Maximal isometric force	3 × 3 × 3 s	3 min	Traditional
	C	8 (M = 6; F = 2)	32	62	168	50	WT	Yes	6	2	12	LL: Squat, lunge with knee lift, arabesque	BW	3 × 30 s	30 s	Traditional
Lundstrom et al. [109]	PL	11 (M = 7; F = 4)	21	66	NR	51	WT	NR	12	1	12	PL: Horizontal jump, vertical jump, frog hop, alternate leg bound, single leg forward hop, lateral cone jump, forward/backward cone jump, squat jump, lunge jump, depth jump, box jump	BW	1–2 × 8–20	1 min	Traditional
	C	11 (M = 6; F = 5)	20	74	NR	54	WT	NR	12	1	12		BW	1–3 × 10–30	1 min	
Mikkola et al. [137]	SL + PL	13 (M = 9; F = 4)	17	63	177	62	HT	NR	8	3	24	PL: Alternate jump, calf jump, squat jump, hurdle jump SL: Half squats, knee extensions, knee flexions, calf raises	Low–load or BW	2–3 × 6–10		Traditional
	C	12 (M = 9; F = 3)	17	60	172	62	HT	NR								
Mikkola et al. [102]	HL	11 (M)	36	78	179	51	MT	Yes	8	2	16	HL: Squat, seated leg press	85–90% 1RM	3 × 4–6	2–3 min	Traditional
	SL + PL	10 (M)	36	79	181	51	MT	Yes	8	2	16	SL: Squat, seated leg press PL: Squat jump, scissor jump	0–40% 1RM	SL: 3 × 6. PL: 2 × 5–10	2–3 min	Complex
	C	6 (M)	34	84	181	48	MT	Yes	8	2	16	LL: Squat, lunges	BW	3 × 40–50 s	10–20 s	Traditional
Millet et al. [103]	HL	7 (M)	24	67	175	70	HT	NR	14	2	28	HL: Hamstring curl, leg press, seated press, squat, leg extension, heel raises	87–93% 1RM	3–5 × 3–5		Traditional
	C	8 (M)	21	65	175	68	HT	NR								
Paavolainen et al. [8]	PL + SL	10 (M)	23	72	179	68	HT	NR	9	2.5	22.5	PL: Alternate jump, bilateral countermovement jump, drop jump, hurdle jump, one-legged SL: Leg press, knee extensor-flexor	PL: BW or barbell. SL: 0–40% 1RM			Traditional
	C	8 (M)	24	70	182	68	HT	NR	–	–	–	LL: Leg exercises	BW	1 × 12		
Pellegrino et al. [12]	PL	11 (M = 7; F = 4)	33	68	172	48	MT	No	6	2.5	15	PL: Squat jump, split scissor jump, double leg bound, alternate leg bound, single leg forward hop, deep knee bend box jump landing, double leg hurdle jump, single leg hurdle hop	BW	2–3 × 8–15		Traditional
	C	11 (M = 7; F = 4)	34	71	171	48	MT	No								
Piacentini et al. [19]	SL	5 (M = 3; F = 2)	45	68	170	NR	MT	NR	6	2	12	SL: Half squat with arm weights, calf exercise (leg press), lunges with arm weights, leg press, eccentric quadriceps contractions, seated calf raises	70% 1RM	3 × 10	2–3 min	Traditional
	HL	6 (M = 4; F = 2)	44	72	170	NR	MT	NR	6	2	12	HL: Half squat with arm weights, calf exercise (leg press), lunges with arm weights, eccentric quadriceps contractions, leg press	85–90% 1RM	4 × 3–4	3–4 min	Traditional
	C	5 (M)	43	71	175	NR	MT	NR								
Saunders et al. [113]	PL	7 (M)	23	68	NR	68	HT	Yes	9	2.5	26	PL: Countermovement jump, knee lift, ankle jump, hamstring curls, alternate leg bound, skip for height, single leg ankle jump, continuous hurdle jump, scissor jump for height SL: Leg press^a^	60% 1RM + BW	1–5 × 6–20		Complex
	C	8 (M)	25	68	NR	70	HT	Yes								
Sedano et al. [115]	SL + PL	6 (M)	24	69	181	69	HT	Yes	12	2	24	SL: Barbell squat, lying leg curl, seated calf raises, leg extension PL: Vertical jump over hurdles, horizontal jump	SL: 70% 1RM. PL: BW	SL: 3 × 7. PL: 10	5 min	Complex
	SL	6 (M)	24	66	179	71	HT	Yes	12	2	24	SL: Barbell squat, lying leg curl, seated calf raises, leg extension	40% 1RM	3 × 20	1 min	Traditional
	C	6 (M)	24	70	185	69	HT	Yes	12	2	24	LL: Squat with band, lying leg curl with band, calf raises with band, leg extension with band	Resistance band	1 × 25	5 min	
Skovgaard et al. [104]	HL	12 (M)	31	77	180	61	WT	No	8	4	32	HL: Squat, deadlift, leg press	2wk 65–67% 1RM; 6wk: HL: 80–90% 1RM	HL: 3 × 8–4	3 min	Traditional
	C	9 (M)	31	77	180	59	WT	No								
Spurrs et al. [107]	PL	8 (M)	25	74.7	178	58	WT	No	6	2.5	15	PL: Squat jump, split scissor jump, double leg bound, alternate leg bound, single leg forward hop, depth jump, double leg hurdle jump, single leg hurdle hop	BW	2–3 × 8–15		Traditional
	C	9 (M)	25	70.2	178	58	WT	No								
Štohanzl et al. [111]	PL	9 (F)	32	69	169	39	MT	No	10	2	20	PL: Calf jump, low skater jump, half squat jump	BW	3–4 × 12–3	2 min	Traditional
	PL	11 (F)	32	71	169	34	MT	No	10	1	10	PL: Calf jump, low skater jump, half squat jump	BW	3–4 × 12–3	2 min	Traditional
	C	11 (F)	32	69	169	37	MT	No								
Støren et al. [99]	HL	8 (M = 4; F = 4)	29	60	171	61	HT	No	8	3	24	HL: Half squat	90% 1RM	4 × 4	3 min	Traditional
	C	9 (M = 5; F = 4)	30	71	179	57	WT	No								
Trowell et al. [119]	SL + PL	14 (M = 8; F = 6)	33	73	173	61	HT	No	10	2	20	PL: Ankle bouncing, hurdle jump (40 cm), high-knee drill or a-skip drill, split squat jump, countermovement jump or drop jump (45 cm) SL: Back squat, single leg deadlift	SL: 70% 1RM. PL: 0–30% BW	SL: 2–5 × 10. PL: 2–5 × many	3 min	Complex
	C	14 (M = 9; F = 5)	34	70	175	66	HT	No	10	2	20	LL: Sit-up, lunge, among others	BW			
Turner et al. [106]	PL	10 (M = 4; F = 6)	31	65	170	50	WT	NR	6	3	18	PL: Vertical jump, one-legged vertical jump, vertical springing jump, split-squat jump, incline jump	BW	1 × 5–15		Traditional
	C	8 (M = 4; F = 4)	27	72	174	54	WT	NR								
Vikmoen et al. [98]	HL	11 (F)	35	62	169	52	WT	No	11	2	22	HL: Half squat, one-legged leg press, standing one-legged hip flexion, ankle plantarflexion	3wk: 75–85% 1RM; 3wk: 80–87% 1RM; 5wk: 85–90% 1RM	3 × 4–10		Traditional
	C	8 (F)	32	66	170	54	WT	No								
Vorup et al. [96]	HL	9 (M)	39	75	181	60	WT	NR	8	2	16	HL: Half squat, leg press, deadlift	1wk: 75% 1RM; 1wk: 80% 1RM; 2wk: 85% 1RM; 4wk: 90% 1RM	1–4 × 10–4	3 min	Traditional
	C	7 (M)	37	74	184	60	WT	NR								

Age (years), *BM* body mass (kg), *C* control, *D* duration (weeks), *Fq* frequency (session/week), *F* female, *G* group, *H* height (cm), *HL* high load training, *HT* highly trained, *ISO* isometric training, *M* male, *min* minute/s, *MT* moderately trained, *MVC* maximal voluntary contraction, *n* sample size, *NR* not reported, *LL* low load, *PL* plyometric training, *PLv* performance level, *s* seconds, *SL* submaximal training, *ST* exp strength training experience, *TS* total sessions, *V*O_2_max (ml/kg/min), *wk* week/s, *WL* well trained, *1 RM* one repetition maximum

^a^SL training was not considered relevant to the training

**Table 3:** Analysis of the studies included in the meta-analysis of running economy

Study	G	n	Running economy						
			Intensity					Mean pre (SD)	Mean post (SD)
			Measurement	Speed reported	U-shaped RE-speed relationship	Relative to AT	Relative to *V*O_2_max (%)		
Ache-Dias et al. [110]	PL	9 (M = 4; F = 5)	kcal/min	9.00 km/h	< 11.50 km/h	≤ AT	65.03	12.84 (1.61)	12.57 (1.99)
	C	9 (M = 4; F = 5)	kcal/min	9.00 km/h	< 11.50 km/h	≤ AT	65.03	12.63 (2.28)	12.31 (2.23)
Albracht & Arampatzis [116]	ISO	13 (M)	J/m/kg	10.80 km/h	< 11.50 km/h	≤ AT		4.25 (0.34)	4.05 (0.32)
			J/m/kg	12.60 km/h	11.50–14.50 km/h	≤ AT		4.27 (0.32)	4.12 (0.31)
	C	13 (M)	J/m/kg	10.80 km/h	< 11.50 km/h	≤ AT		4.11 (0.32)	4.11 (0.28)
			J/m/kg	12.60 km/h	11.50–14.50 km/h	≤ AT		4.18 (0.34)	4.19 (0.30)
Berryman et al. [114]	SL	12 (M)	ml/kg/km	12.00 km/h	11.50–14.50 km/h		73.11	207.00 (15.00)	199.00 (12.00)
	PL	11 (M)	ml/kg/km	12.00 km/h	11.50–14.50 km/h		73.11	218.00 (16.00)	203.00 (13.00)
	C	5 (M)	ml/kg/km	12.00 km/h	11.50–14.50 km/h		73.11	199.00 (18.00)	199.00 (20.00)
Blagrove et al. [118]	SL + PL	9 (M = 4; F = 5)	kJ/kg^0.67^/km	sLTP	11.50–14.50 km/h	≤ AT	83.53	18.70 (1.30)	18.10 (1.40)
			kJ/kg^0.67^/km	sLTP—1.00 km/h	11.50–14.50 km/h	≤ AT	77.75	18.50 (1.30)	18.30 (0.90)
			kJ/kg^0.67^/km	sLTP—2.00 km/h	11.50–14.50 km/h	≤ AT	71.97	18.80 (1.20)	18.10 (1.50)
	C	9 (M = 4; F = 5)	kJ/kg^0.67^/km	sLTP	11.50–14.50 km/h	≤ AT	83.53	18.50 (1.30)	18.30 (0.80)
			kJ/kg^0.67^/km	sLTP—1.00 km/h	11.50–14.50 km/h	≤ AT	77.75	19.20 (1.40)	18.50 (1.60)
			kJ/kg^0.67^/km	sLTP—2.00 km/h	11.50–14.50 km/h	≤ AT	71.97	18.80 (1.30)	18.70 (1.20)
Damasceno et al. [101]	HL	9 (M)	ml/kg/min	12.00 km/h	11.50–14.50 km/h	≤ AT	76.57	42.50 (3.10)	41.90 (4.00)
	C	9 (M)	ml/kg/min	12.00 km/h	11.50–14.50 km/h	≤ AT	76.57	41.80 (4.60)	41.00 (4.20)
do Carmo et al. [108]	PL	15 (M)	ml/kg/min	Avg 11.00 to 12.00 km/h	11.50–14.50 km/h	≤ AT	73.95	226.05 (18.40)	215.90 (17.70)
	C	13 (M)	ml/kg/min	Avg 11.00 to 12.00 km/h	11.50–14.50 km/h	≤ AT	73.95	224.90 (18.00)	224.90 (19.90)
Ferrauti et al. [97]	HL	11 (M = 9; F = 2)	ml/kg/min	10.08 km/h	< 11.50 km/h	≤ AT	71.77	36.90 (2.10)	37.70 (2.90)
			ml/kg/min	8.64 km/h	< 11.50 km/h	≤ AT	62.56	31.70 (1.90)	33.30 (3.10)
	C	11 (M = 7; F = 4)	ml/kg/min	10.08 km/h	< 11.50 km/h	≤ AT	71.77	37.10 (3.10)	38.80 (2.80)
			ml/kg/min	8.64 km/h	< 11.50 km/h	≤ AT	62.56	32.80 (4.30)	34.1 (3.00)
Fletcher et al. [117]	ISO	6 (M)	kJ/kg/km	95% sLT	11.50–14.50 km/h	≤ AT		4.46 (0.16)	4.44 (0.21)
				75% sLT	11.50–14.50 km/h	≤ AT		4.17 (0.33)	4.21 (0.27)
				85% sLT	11.50–14.50 km/h	≤ AT		4.27 (0.18)	4.26 (0.28)
	C	6 (M)	kJ/kg/km	95% sLT	11.50–14.50 km/h	≤ AT		4.64 (0.16)	4.63 (0.16)
				75% sLT	11.50–14.50 km/h	≤ AT		4.49 (0.22)	4.50 (0.21)
				85% sLT	11.50–14.50 km/h	≤ AT		4.64 (0.16)	4.63 (0.16)
Johnston et al. [136]	HL + SL	6 (F)	ml/kg/min	13.80 km/h	11.50–14.50 km/h		85.59	44.50 (1.00)	42.80 (1.10)
				12.84 km/h	11.50–14.50 km/h		79.80	41.60 (1.10)	39.90 (0.80)
	C	6 (F)	ml/kg/min	13.80 km/h	11.50–14.50 km/h		85.59	42.80 (0.70)	43.20 (0.70)
				12.84 km/h	11.50–14.50 km/h		79.80	39.80 (0.50)	40.00 (0.70)
Kelly et al. [100]	HL	7 (F)	ml/kg/min	3 km race pace	< 11.50 km/h		71.91	27.50 (3.60)	29.30 (2.60)
	C	9 (F)	ml/kg/min	3 km race pace	< 11.50 km/h		71.91	29.60 (4.60)	30.20 (7.00)
Li et al. [20]	HL + PL	10 (M)	ml/kg/min	16.00 km/h	> 14.50 km/h	≤ AT	91.67	60.77 (4.17)	56.09 (2.55)
			ml/kg/min	12.00 km/h	11.50–14.50 km/h	≤ AT	72.08	46.62 (3.2)	44.20 (2.32)
			ml/kg/min	14.00 km/h	11.50–14.50 km/h	≤ AT	81.61	54.45 (3.18)	50.17 (3.04)
	HL	9 (M)	ml/kg/min	16.00 km/h	> 14.50 km/h	≤ AT	91.67	59.60 (3.04)	57.88 (4.11)
			ml/kg/min	12.00 km/h	11.50–14.50 km/h	≤ AT	72.08	47.01 (3.14)	45.06 (3.82)
			ml/kg/min	14.00 km/h	11.50–14.50 km/h	≤ AT	81.61	53.41 (3.26)	50.78 (4.07)
	C	9 (M)	ml/kg/min	16.00 km/h	> 14.50 km/h	≤ AT	91.67	61.00 (3.00)	61.35 (1.79)
			ml/kg/min	12.00 km/h	11.50–14.50 km/h	≤ AT	72.08	47.81 (3.24)	45.71 (3.05)
			ml/kg/min	14.00 km/h	11.50–14.50 km/h	≤ AT	81.61	53.95 (2.27)	52.33 (2.31)
Luckin-Baldwin et al. [135]	SL + HL	14 (M = 10; F = 4)	ml/kg^0.75^/min	Different speeds				665.48 (53.81)	633.14 (59.37)
	C	11 (M = 9; F = 2)	ml/kg^0.75^/min	Different speeds				685.84 (45.18)	670.42 (65.78)
Lum et al. [105]	PL	7 (M)	ml/kg/min	12.00 km/h	11.50–14.50 km/h	≤ AT	83.47	45.30 (1.80)	44.60 (2.30)
			ml/kg/min	10.00 km/h	< 11.50 km/h	≤ AT	70.54	37.90 (2.10)	36.80 (1.70)
	C	7 (M)	ml/kg/min	12.00 km/h	11.50–14.50 km/h	≤ AT	83.47	45.10 (3.40)	44.70 (4.10)
			ml/kg/min	10.00 km/h		≤ AT	70.54	38.50 (3.30)	37.30 (3.10)
Lum et al. [112]	PL	9 (M = 6; F = 3)	J/kg/km	F: 10.00 km/h; M: 12.00 km/h	11.50–14.50 km/h		69.40	1.08 (0.06)	1.07 (0.06)
			J/kg/km	F: 12.00 km/h; M: 14.00 km/h	11.50–14.50 km/h		82.02	1.09 (0.07)	1.08 (0.07)
	ISO	9 (M = 6; F = 3)	J/kg/km	F: 10.00 km/h; M: 12.00 km/h	11.50–14.50 km/h		69.40	1.06 (0.06)	1.04 (0.08)
			J/kg/km	F: 12.00 km/h; M: 14.00 km/h	11.50–14.50 km/h		82.02	1.08 (0.10)	1.04 (0.09)
	C	8 (M = 6; F = 2)	J/kg/km	F: 10.00 km/h; M: 12.00 km/h	11.50–14.50 km/h		69.40	1.13 (0.12)	1.11 (0.07)
			J/kg/km	F: 12.00 km/h; M: 14.00 km/h	11.50–14.50 km/h		82.02	1.12 (0.12)	1.10 (0.06)
Lundstrom et al. [109]	PL	11 (M = 7; F = 4)	ml/kg/min	Avg values over incremental test				216.30 (17.90)	200.20 (29.50)
	C	11 (M = 6; F = 5)	ml/kg/min	Avg values over incremental test				218.60 (19.40)	192.90 (37.00)
Mikkola et al. [137]	SL + PL	13 (M = 9; F = 4)	ml/kg/min	14.00 km/h	11.50–14.50 km/h		83.01	52.10 (3.10)	50.60 (3.60)
			ml/kg/min	10.00 km/h	< 11.50 km/h		61.43	38.20 (2.80)	38.70 (3.00)
			ml/kg/min	12.00 km/h	11.50–14.50 km/h		72.54	45.00 (3.10)	44.10 (3.30)
			ml/kg/min	13.00 km/h	11.50–14.50 km/h		77.05	48.10 (3.20)	47.40 (3.30)
	C	12 (M = 9; F = 3)	ml/kg/min	14.00 km/h	11.50–14.50 km/h		83.01	51.00 (4.30)	52.30 (3.90)
			ml/kg/min	10.00 km/h	< 11.50 km/h		61.43	38.10 (3.10)	38.90 (3.00)
			ml/kg/min	12.00 km/h	11.50–14.50 km/h		72.54	45.10 (3.70)	45.90 (3.90)
			ml/kg/min	13.00 km/h	11.50–14.50 km/h		77.05	47.60 (4.10)	49.00 (3.40)
Mikkola et al. [102]	HL	11 (M)	ml/kg/min	12.00 km/h	11.50–14.50 km/h	> AT	86.06	218.00 (10.00)	219.00 (11.00)
			ml/kg/min	10.00 km/h	< 11.50 km/h	≤ AT	74.07	224.00 (10.00)	225.00 (11.00)
	SL + PL	10 (M)	ml/kg/min	12.00 km/h	11.50–14.50 km/h	> AT	86.06	212.00 (17.00)	210.00 (16.00)
			ml/kg/min	10.00 km/h	< 11.50 km/h	≤ AT	74.07	217.00 (18.00)	217.00 (20.00)
	C	6 (M)	ml/kg/min	12.00 km/h	11.50–14.50 km/h	> AT	86.06	208.00 (15.00)	206.00 (18.00)
			ml/kg/min	10.00 km/h	< 11.50 km/h	≤ AT	74.07	216.00 (14.00)	217.00 (22.00)
Millet et al. [103]	HL	7 (M)	ml/kg/km	75% s*V*O_2_max	> 14.50 km/h	≤ AT	75.00	193.60 (4.30)	180.30 (20.00)
			ml/kg/km	92% s*V*O_2_max	> 14.50 km/h	> AT	92.00	196.40 (5.50)	185.40 (16.30)
	C	8 (M)	ml/kg/km	75% s*V*O_2_max	> 14.50 km/h	≤ AT	75.00	189.80 (13.10)	203.20 (20.20)
			ml/kg/km	92% s*V*O_2_max	> 14.50 km/h	> AT	92.00	194.60 (22.30)	205.20 (18.10)
Paavolainen et al. [8]	PL + SL	10 (M)	ml/kg/min	15.01 km/h	> 14.50 km/h	> AT	78.17	51.80 (1.20)	47.60 (1.30)
	C	8 (M)	ml/kg/min	15.01 km/h	> 14.50 km/h	> AT	78.17	50.70 (1.50)	51.40 (2.10)
Pellegrino et al. [12]	PL	11 (M = 7; F = 4)	J/kg/min	12.10 km/h	11.50–14.50 km/h	≤ AT		927.00 (28.00)	920.00 (29.00)
			J/kg/min	7.74 km/h	< 11.50 km/h	≤ AT		646.00 (17.00)	643.00 (20.00)
			J/kg/min	9.18 km/h	< 11.50 km/h	≤ AT		703.00 (17.00)	696.00 (18.00)
			J/kg/min	10.62 km/h	< 11.50 km/h	≤ AT		789.00 (19.00)	779.00 (28.00)
			J/kg/min	13.53 km/h	11.50–14.50 km/h	> AT		1070.00 (39.00)	1095.00 (35.00)
			J/kg/min	14.97 km/h	> 14.50 km/h	> AT		1220.00 (50.00)	1217.00 (34.00)
			J/kg/min	16.41 km/h	> 14.50 km/h	> AT		1337.00 (84.00)	1415.00 (52.00)
	C	11 (M = 7; F = 4)	J/kg/min	12.10 km/h	11.50–14.50 km/h	≤ AT		998.00 (30.00)	991.00 (31.00)
			J/kg/min	7.74 km/h	< 11.50 km/h	≤ AT		673.00 (17.00)	685.00 (20.00)
			J/kg/min	9.18 km/h	< 11.50 km/h	≤ AT		746.00 (17.00)	763.00 (18.00)
			J/kg/min	10.62 km/h	< 11.50 km/h	≤ AT		857.00 (19.00)	882.00 (28.00)
			J/kg/min	13.53 km/h	11.50–14.50 km/h	> AT		1163.00 (41.00)	1131.00 (37.00)
			J/kg/min	14.97 km/h	> 14.50 km/h	> AT		1383.00 (59.00)	1340.00 (41.00)
			J/kg/min	16.41 km/h	> 14.50 km/h	> AT		1518.00 (168.00)	1451.00 (105.00)
Piacentini et al. [19]	SL	5 (M = 3; F = 2)	ml/kg/min	Avg 11.75 km/h	11.50–14.50 km/h			42.30 (7.40)	42.20 (8.20)
			ml/kg/min	Avg 9.75 km/h	< 11.50 km/h	≤ AT		35.30 (6.20)	34.90 (7.00)
			ml/kg/min	Avg 10.75 km/h	< 11.50 km/h	≤ AT		39.20 (6.90)	38.00 (7.10)
	HL	6 (M = 4; F = 2)	ml/kg/min	Avg 11.75 km/h	11.50–14.50 km/h			42.80 (5.00)	44.20 (4.00)
			ml/kg/min	Avg 9.75 km/h	< 11.50 km/h	≤ AT		37.00 (5.20)	36.20 (4.20)
			ml/kg/min	Avg 10.75 km/h	< 11.50 km/h	≤ AT		42.10 (4.00)	39.50 (3.50)
	C	5 (M)	ml/kg/min	Avg 11.75 km/h	11.50–14.50 km/h			43.40 (3.60)	42.90 (40)
			ml/kg/min	Avg 9.75 km/h	< 11.50 km/h	≤ AT		34.70 (2.40)	34.60 (2.30)
			ml/kg/min	Avg 10.75 km/h	< 11.50 km/h	≤ AT		39.50 (2.80)	39.20 (0.80)
Saunders et al. [113]	PL	7 (M)	J/kg/min	16.00 km/h	> 14.50 km/h	≤ AT	81.04	1190.80 (87.20)	1162.80 (116.10)
			J/kg/min	18.00 km/h	> 14.50 km/h	> AT	90.96	1539.20 (292.20)	1481.40 (216.60)
	C	8 (M)	J/kg/min	16.00 km/h	> 14.50 km/h	≤ AT	81.04	1209.40 (41.90)	1138.60 (133.60)
			J/kg/min	18.00 km/h	> 14.50 km/h	> AT	90.96	1528.20 (256.60)	1435.70 (176.90)
Sedano et al. [115]	SL + PL	6 (M)	ml/kg/min	16.00 km/h	> 14.50 km/h		74.64	50.92 (0.89)	49.37 (1.03)
			ml/kg/min	12.00 km/h	11.50–14.50 km/h		55.98	38.69 (1.05)	37.25 (0.75)
			ml/kg/min	14.00 km/h	> 14.50 km/h		65.31	44.80 (1.11)	42.48 (1.05)
	SL	6 (M)	ml/kg/min	16.00 km/h	> 14.50 km/h		74.64	50.60 (1.27)	49.84 (1.53)
			ml/kg/min	12.00 km/h	11.50–14.50 km/h		55.98	38.92 (1.41)	37.92 (1.50)
			ml/kg/min	14.00 km/h	> 14.50 km/h		65.31	45.40 (1.09)	44.30 (1.19)
	C	6 (M)	ml/kg/min	16.00 km/h	> 14.50 km/h		74.64	50.67 (0.95)	50.69 (0.94)
			ml/kg/min	12.00 km/h	11.50–14.50 km/h		55.98	38.66 (1.22)	38.59 (1.02)
			ml/kg/min	14.00 km/h	> 14.50 km/h		65.31	46.95 (2.82)	46.88 (2.14)
Skovgaard et al. [104]	HL	12 (M)	ml/kg/km	12.00 km/h	11.50–14.50 km/h		62.71	195.00 (4.00)	189.00 (4.00)
	C	9 (M)	ml/kg/km	12.00 km/h	11.50–14.50 km/h		62.71	180.00 (4.00)	178.00 (6.00)
Spurrs et al. [107]	PL	8 (M)	ml/kg/min	14.00 km/h	11.50–14.50 km/h	≤ AT	55.43	33.35 (5.15)	32.23 (4.27)
			ml/kg/min	12.00 km/h	11.50–14.50 km/h	≤ AT	43.44	26.05 (4.11)	24.30 (3.68)
			ml/kg/min	16.00 km/h	> 14.50 km/h	> AT	69.84	41.96 (6.14)	40.22 (5.43)
	C	9 (M)	ml/kg/min	14.00 km/h	11.50–14.50 km/h	≤ AT	55.43	30.62 (3.29)	30.46 (3.98)
			ml/kg/min	12.00 km/h	11.50–14.50 km/h	≤ AT	43.44	24.08 (2.87)	24.21 (3.37)
			ml/kg/min	16.00 km/h	> 14.50 km/h	> AT	69.84	38.64 (4.95)	38.85 (5.33)
Štohanzl et al. [111]	PL	9 (F)	ml/kg/min	sVT_2_	< 11.50 km/h	≤ AT	80.74	28.90 (2.70)	28.40 (2.60)
			ml/kg/min	7.00 km/h	< 11.50 km/h	≤ AT	73.77	28.10 (2.50)	26.80 (3.10)
			ml/kg/min	9.00 km/h	< 11.50 km/h	> AT	87.30	33.10 (2.70)	32.10 (3.10)
	PL	11 (F)	ml/kg/min	sVT_2_	< 11.50 km/h	≤ AT	83.88	31.20 (3.50)	30.80 (3.50)
			ml/kg/min	7.00 km/h	< 11.50 km/h	≤ AT	68.99	24.60 (4.20)	23.60 (4.90)
			ml/kg/min	9.00 km/h	< 11.50 km/h	≤ AT	81.69	29.00 (5.00)	28.60 (5.30)
	C	11 (F)	ml/kg/min	sVT_2_	< 11.50 km/h	≤ AT	83.88	30.20 (3.40)	31.60 (3.80)
			ml/kg/min	7.00 km/h	< 11.50 km/h	≤ AT	68.99	25.90 (1.90)	25.30 (2.40)
			ml/kg/min	9.00 km/h	< 11.50 km/h	≤ AT	81.69	30.80 (2.20)	30.50 (1.90)
Støren et al. [99]	HL	8 (M = 4; F = 4)	ml/kg^0.75^/min	70% s*V*O_2_max		≤ AT	70.00	0.68 (0.04)	0.65 (0.03)
	C	9 (M = 5; F = 4)	ml/kg^0.75^/min	70% s*V*O_2_max		≤ AT	70.00	0.68 (0.05)	0.69 (0.05)
Trowell et al. [119]	SL + PL	14 (M = 8; F = 6)	kJ/kg/min	12.00 km/h	11.50–14.50 km/h	≤ AT	66.23	4.51 (0.40)	4.50 (0.33)
	C	14 (M = 9; F = 5)	kJ/kg/min	12.00 km/h	11.50–14.50 km/h	≤ AT	66.23	4.43 (0.41)	4.35 (0.37)
Turner et al. [106]	PL	10 (M = 4; F = 6)	m/ml/kg	11.27 km/h	< 11.50 km/h			5.20 (0.34)	5.32 (0.39)
			m/ml/kg	9.65 km/h	< 11.50 km/h			5.14 (0.36)	5.30 (0.36)
	C	8 (M = 4; F = 4)	m/ml/kg	11.27 km/h	< 11.50 km/h			5.07 (0.42)	5.04 (0.45)
			m/ml/kg	9.65 km/h	< 11.50 km/h			5.21 (0.37)	5.12 (0.28)
Vikmoen et al. [98]	HL	11 (F)	ml/kg/min	10.00 km/h	< 11.50 km/h	≤ AT	85.74	45.83 (1.65)	46.13 (1.73)
	C	8 (F)	ml/kg/min	10.00 km/h	< 11.50 km/h	≤ AT	85.74	45.39 (1.48)	45.35 (1.40)
Vorup et al. [96]	HL	9 (M)	ml/kg/min	Avg 15.00 km/h	> 14.50 km/h	> AT	81.74	54.70 (6.00)	52.50 (4.60)
			ml/kg/min	Avg 11.00 km/h	< 11.50 km/h	≤ AT	59.95	42.40 (4.80)	40.30 (2.70)
	C	7 (M)	ml/kg/min	Avg 15.00 km/h	> 14.50 km/h	> AT	81.74	53.10 (4.20)	53.60 (6.10)
			ml/kg/min	Avg 11.00 km/h	< 11.50 km/h	≤ AT	59.95	42.30 (1.90)	42.30 (3.70)

*avg* average, *AT* anaerobic threshold, *C* control, *F* female, *G* group, *HL* high load training, *ISO* isometric training, *M* male, *n* sample size, *PL* plyometric training, *SD* standard deviation, *SL* submaximal load training, *sLT* speed at lactate threshold, *sLTP* speed at lactate turn point, *sVT*_*2*_ speed at VT_2_, *sVO*_*2*_*max* speed at *V*O_2_max

### Risk of Bias, Publication Bias and Certainty Assessment

The median of risk of bias was 6 of 7 [ranging from 5 to 7; moderate to low risk of bias; Table S2 (OSM)]. No publication bias was found in any of the analyses [Fig. S1 (OSM)]. We found a moderate and low level of certainty of evidence, due to an analysis with moderate risk of bias in the combined methods group, and low number of participants included in the analyses and/or the confidence interval crossed the small effect size in all main analyses (Table 4).

**Table 4:** GRADE assessment for the certainty of evidence

Certainty assessment						No. of participants		Certainty
No. of studies	Risk of bias	Inconsistency	Indirectness	Imprecision	Publication bias	Strength training	Control group	
High load training (follow-up: mean 8.6 weeks)								
11	Not serious	Not serious	Not serious	Serious^a^	Undetected	108	98	⨁⨁⨁◯ Moderate
Submaximal training (follow-up: mean 8.7 weeks)								
3	Not serious	Not serious	Not serious	Serious^a^	Undetected	23	16	⨁⨁⨁◯ Moderate
Plyometric training (follow-up: mean 7.9 weeks)								
11	Not serious	Not serious	Not serious	Serious^a^	Undetected	118	100	⨁⨁⨁◯ Moderate
Isometric training (follow-up: mean 9.3 weeks)								
3	Not serious	Not serious	Not serious	Serious^a^	Undetected	28	27	⨁⨁⨁◯ Moderate
Combined methods training (follow-up: mean 11.5 weeks)								
9	Serious^b^	Not serious	Not serious	Serious^a^	Undetected	82	73	⨁⨁◯◯ Low

^a^Downgraded by one level because n < 800 and/or the 95% confidence interval crossed the small effect size

^b^Downgraded by one level because the median PEDro scale score was < 6

### Main Effects

Thirty-one studies (involving 37 experimental-control comparisons) measured RE at speeds between 7.00 and 18.00 km/h, providing a total of 80 ES for analysis.

**Fig. 2:**
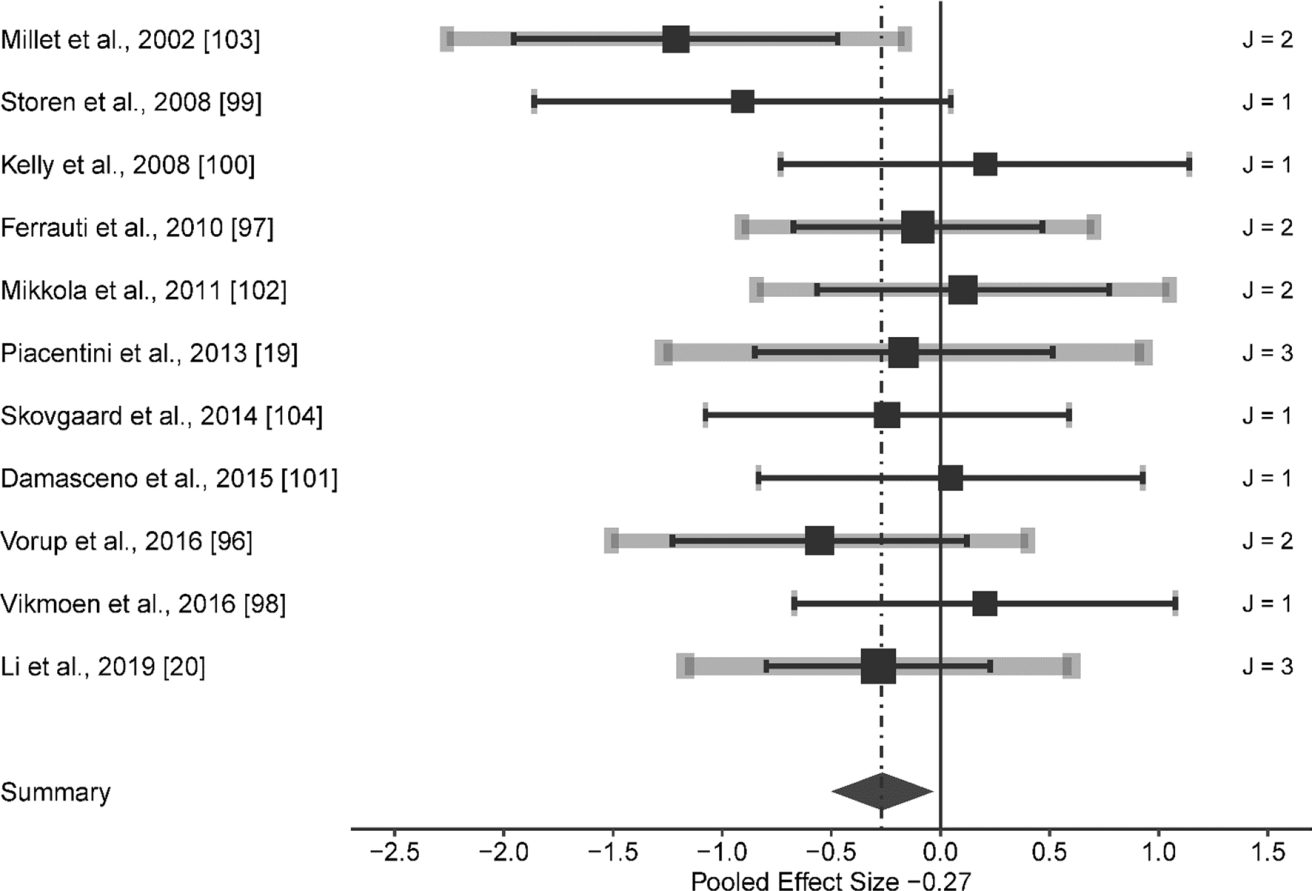
Forest plots of the included studies for high load training and its effect on running economy. The black squares represent the mean observed effect size of the study, the size of square represent the weight of the study and the black lines represent the 95% confidence interval. The grey line represents a 95% confidence interval based on the sampling variance of individual observed effect sizes in a study, and its thickness is proportional to the number of effect sizes reported within studies. *J* number of effect sizes within studies

**Fig. 3:**
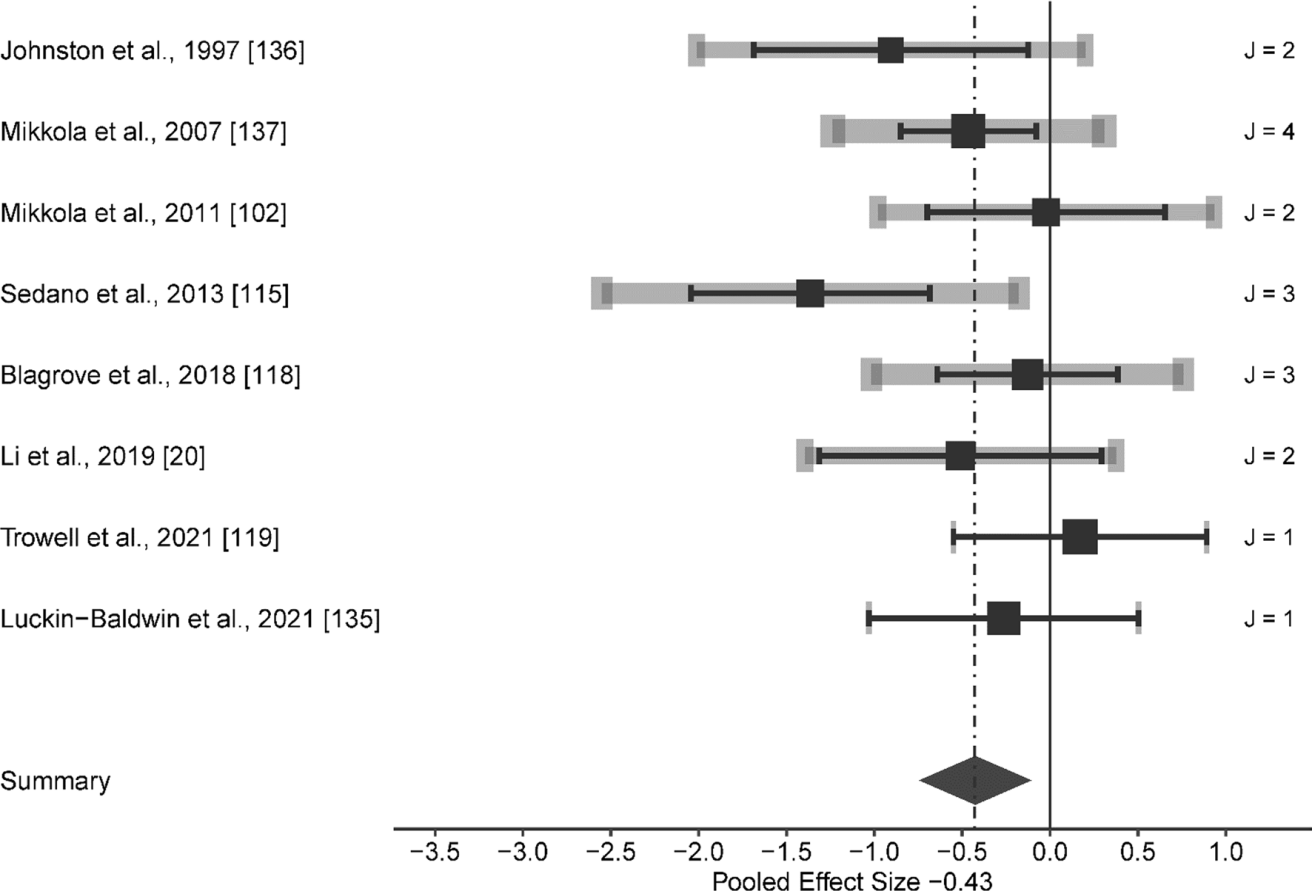
Forest plots of the included studies for combined methods training and its effect on running economy. The black squares represent the mean observed effect size of the study, the size of square represent the weight of the study and the black lines represent the 95% confidence interval. The grey line represents a 95% confidence interval based on the sampling variance of individual observed effect sizes in a study, and its thickness is proportional to the number of effect sizes reported within studies. *J* number of effect sizes within studies

Compared to the control group, the HL exerted a small significant effect on RE at speeds ranging from 8.64 to 17.85 km/h (ES [95% CI] = − 0.266 [− 0.516 to − 0.015], *p* = 0.039; *Q*(18) = 16.816, *p* = 0.536, LRT_level2_ = 0, *p* = 1, LRT_level3_ = 0.089, *p* = 0.765, Fig. 2). Combined methods group induced a moderate significant effect, although with significant heterogeneity (ES [95% CI] = − 0.647 [− 1.140 to − 0.154], *p* = 0.013; *Q*(19) = 40.696, *p* = 0.003, LRT_level2_ = 0.033, *p* = 0.855, LRT_level3_ = 2.569, *p* = 0.109). After removing outlier groups (i.e., Paavolainen et al. [8] and the 16.00 km/h group in Li et al. [20]), the main effect remained moderate significant on RE (i.e., from 10.00 to 14.45 km/h), although without significant heterogeneity (ES [95% CI] = − 0.426 [− 0.768 to − 0.083], *p* = 0.018, *Q*(17) = 19.3, *p* = 0.312, LRT_level2_ = 0, *p* = 1, LRT_level3_ = 1.671, *p* = 0.196, Fig. 3).

**Fig. 4:**
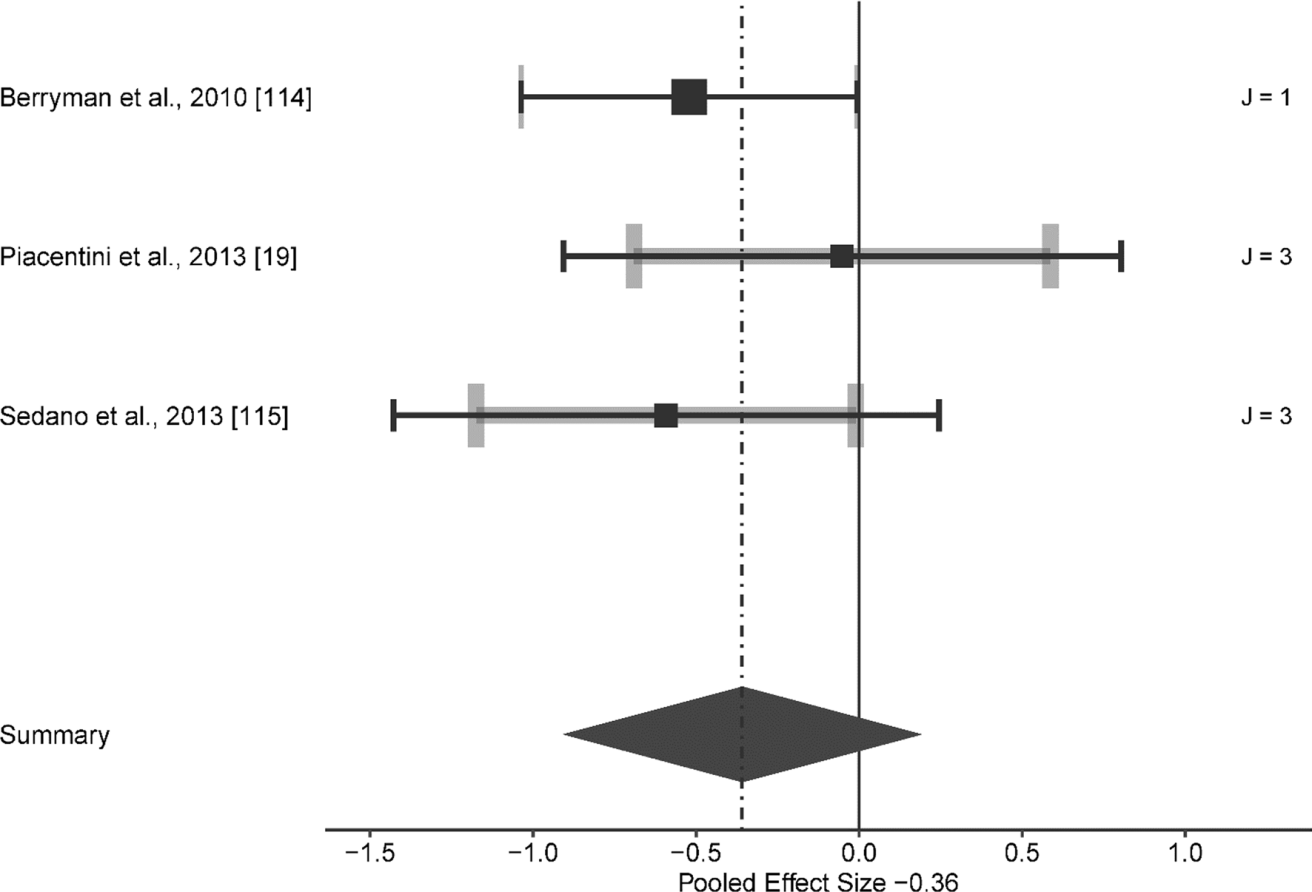
Forest plots of the included studies for submaximal load training and its effect on running economy. The black squares represent the mean observed effect size of the study, the size of square represent the weight of the study and the black lines represent the 95% confidence interval. The grey line represents a 95% confidence interval based on the sampling variance of individual observed effect sizes in a study, and its thickness is proportional to the number of effect sizes reported within studies. *J* number of effect sizes within studies

**Fig. 5:**
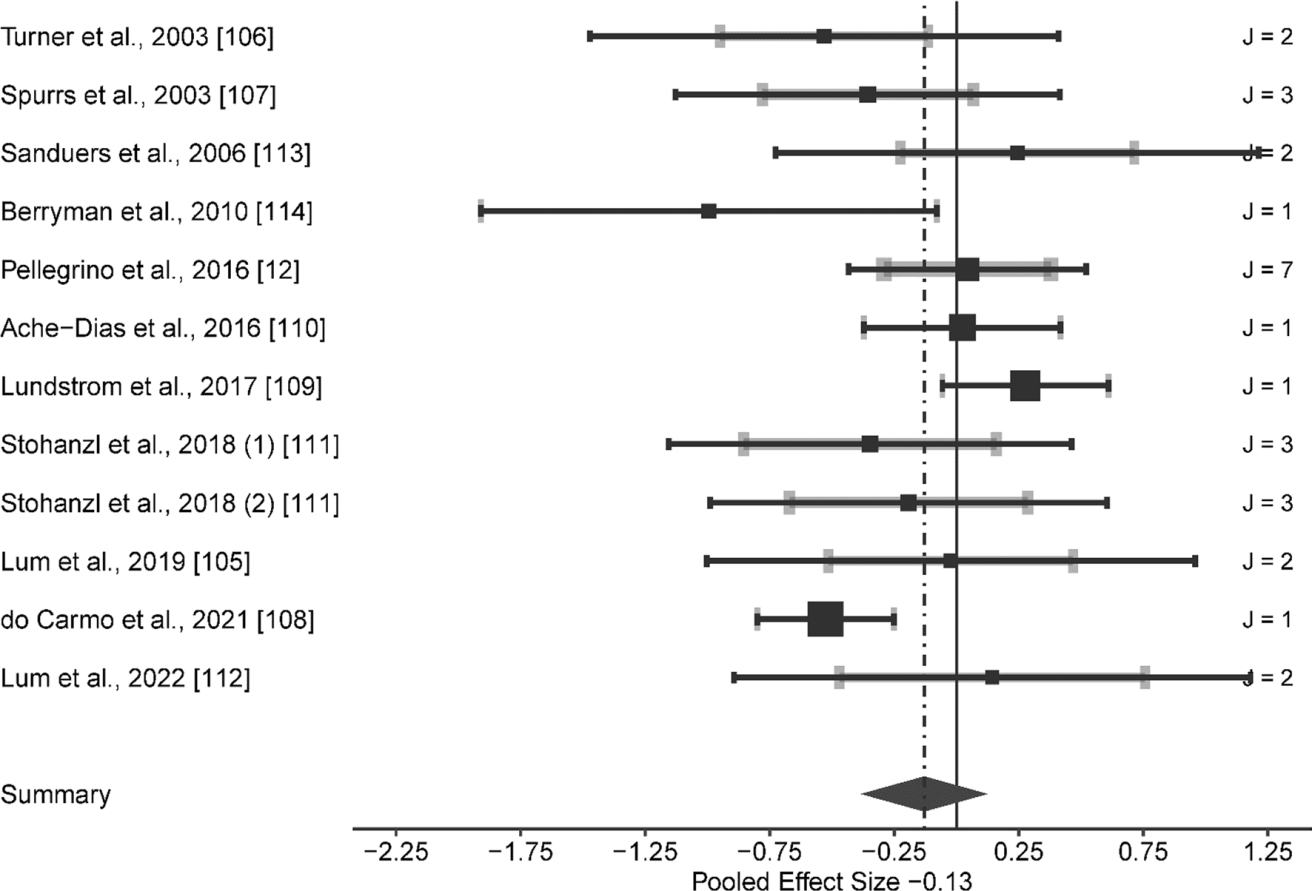
Forest plots of the included studies for plyometric training and its effect on running economy. The black squares represent the mean observed effect size of the study, the size of square represent the weight of the study and the black lines represent the 95% confidence interval. The grey line represents a 95% confidence interval based on the sampling variance of individual observed effect sizes in a study, and its thickness is proportional to the number of effect sizes reported within studies. *J* number of effect sizes within studies

**Fig. 6:**
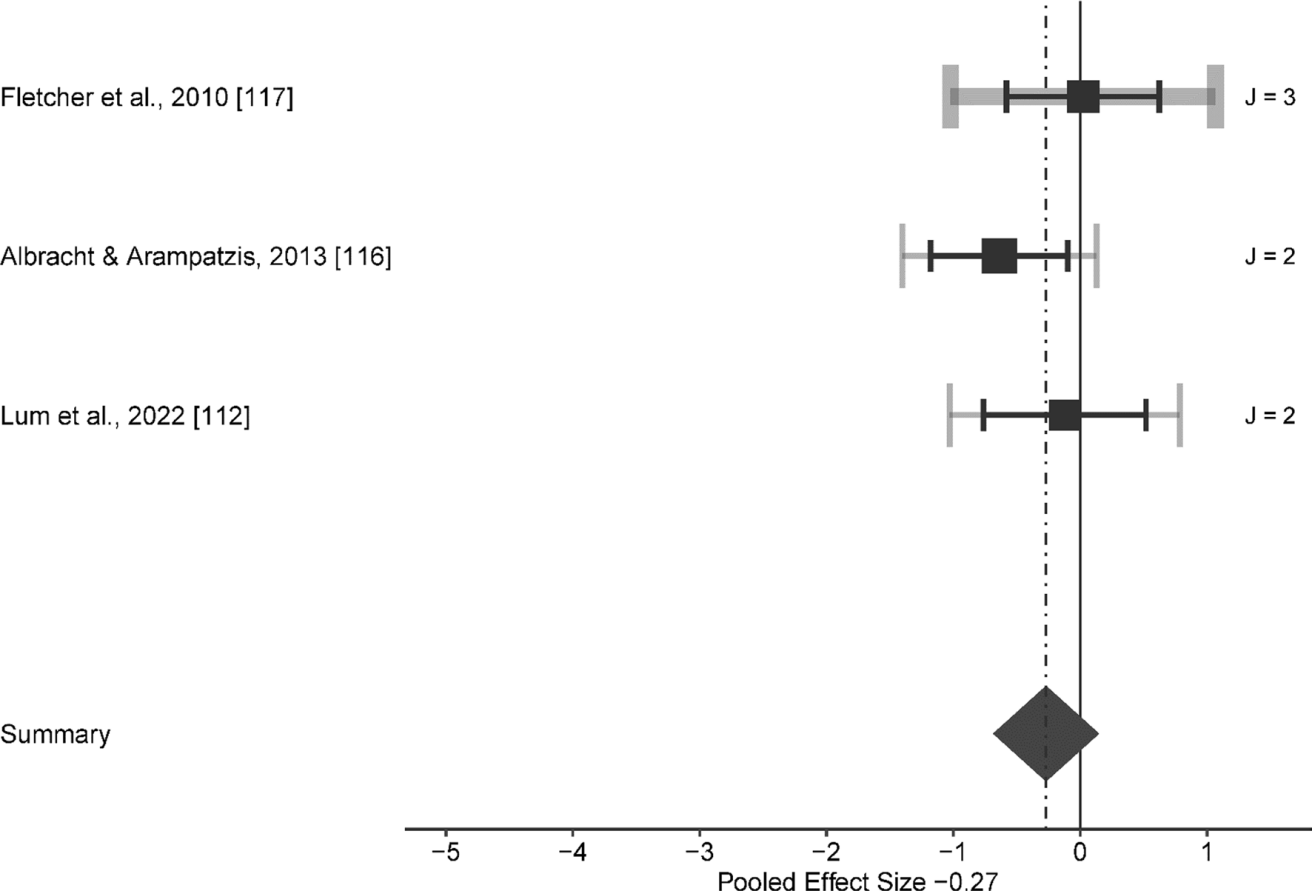
Forest plots of the included studies for isometric training and its effect on running economy. The black squares represent the mean observed effect size of the study, the size of square represent the weight of the study and the black lines represent the 95% confidence interval. The grey line represents a 95% confidence interval based on the sampling variance of individual observed effect sizes in a study, and its thickness is proportional to the number of effect sizes reported within studies. *J* number of effect sizes within studies

Compared to the control condition, no significant effect was found for SL at speeds between 9.75 to 16.00 km/h (ES [95% CI] = − 0.365 [− 0.875 to 0.146], *p* = 0.131, *Q*(6) = 1.607, *p* = 0.952, LRT_level2_ = 0, *p* = 1, LRT_level3_ = 0, *p* = 1, Fig. 4), for PL at speeds ranging from 7.00 to 18.00 km/h (ES [95% CI] = − 0.122 [− 0.299 to 0.054], *p* = 0.167, *Q*(27) = 16.855, *p* = 0.935, LRT_level2_ = 0, *p* = 1, LRT_level3_ < 0.001, *p* = 0.999, Fig. 5) and for ISO at speed between 10.00 to 15.28 km/h. ES [95% CI] = − 0.269 [− 0.79 to 0.252], *p* = 0.253, *Q* (6) = 3.276, *p* = 0.774, LRT_level2_ = 0, *p* = 1, LRT_level3_ = 0.211, *p* = 0.646, Fig. 6).

### Sub-group and Meta-regression Analysis

**Fig. 7:**
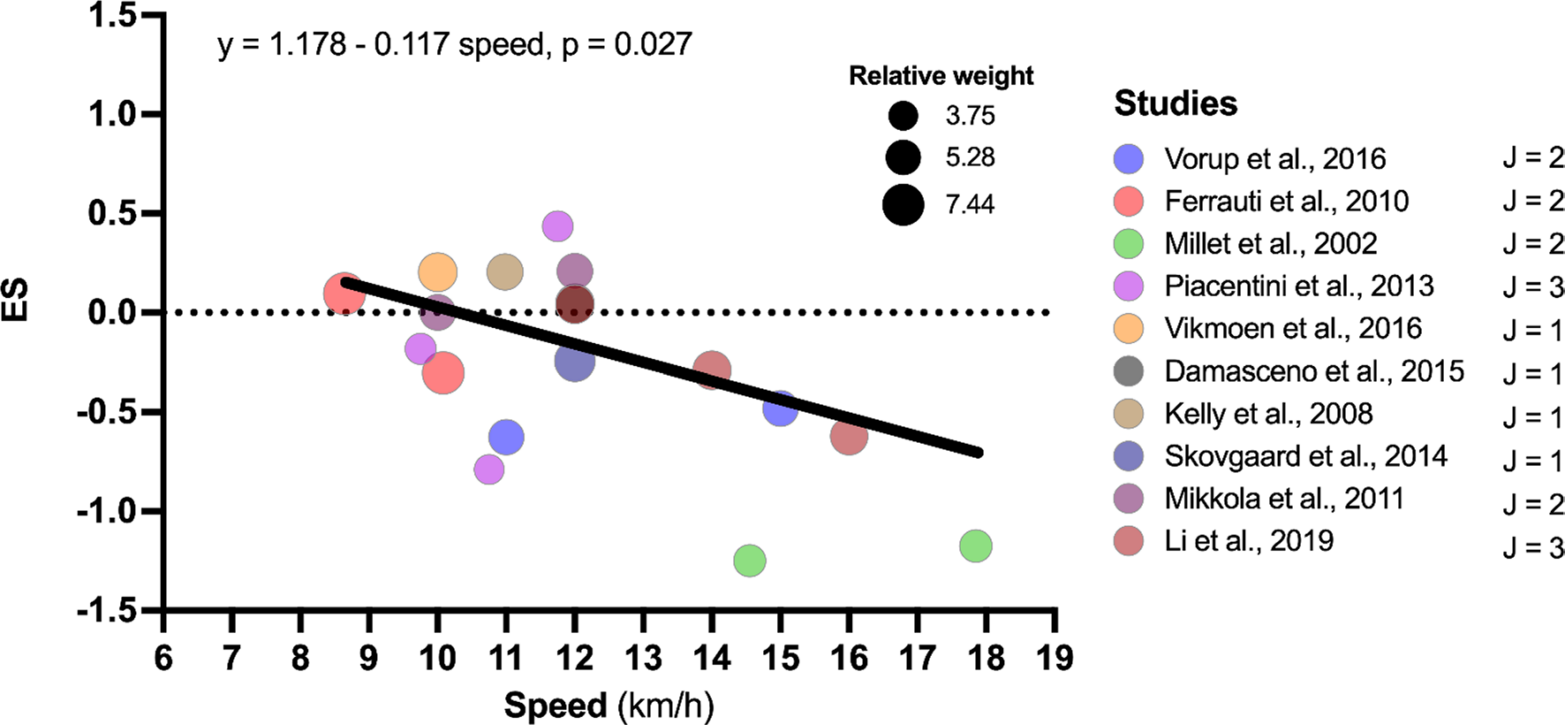
Meta-regression analysis for the effect of absolute speed (continuous) on running economy effect size in high load training. *ES* effect size, *J* number of effect sizes within studies

**Fig. 8:**
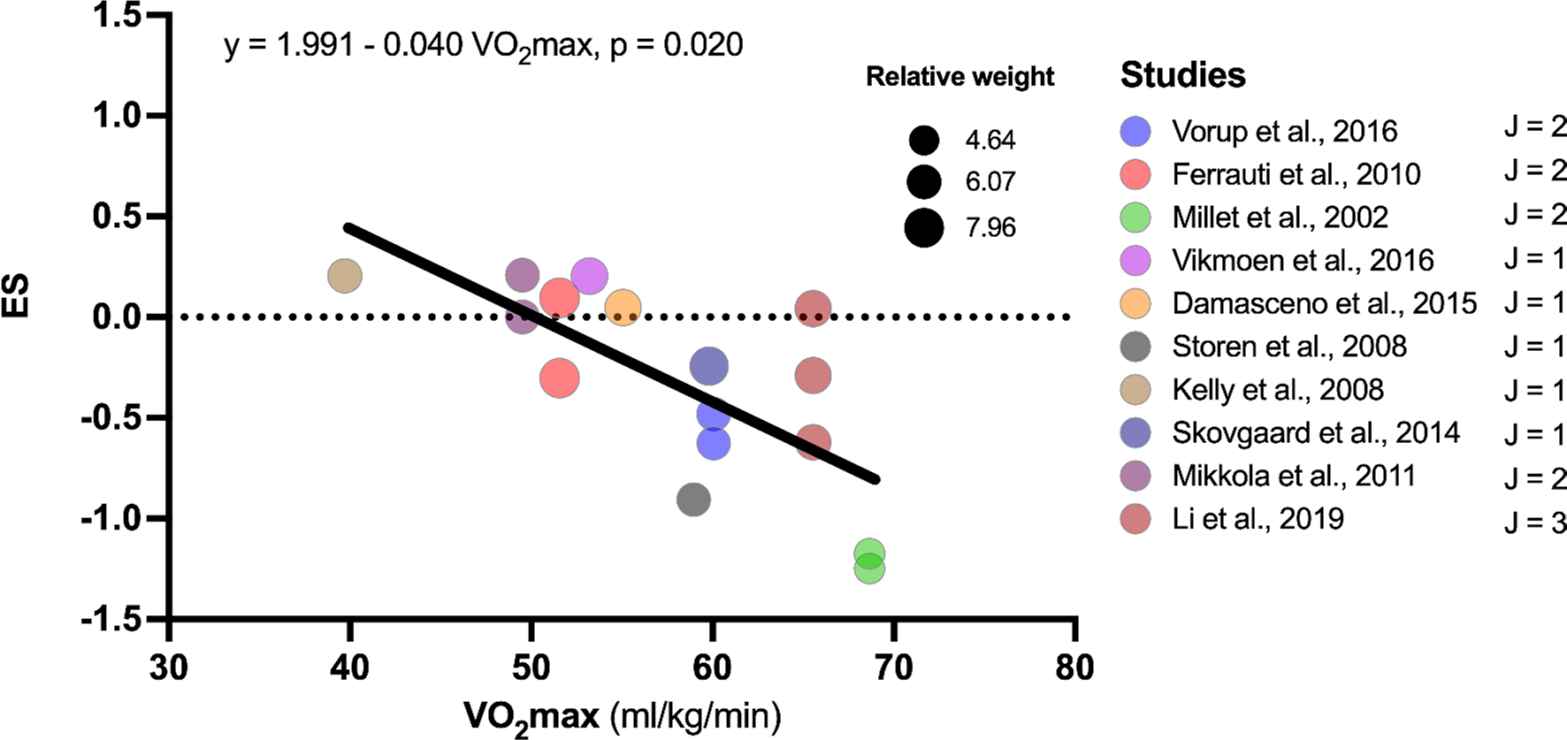
Meta-regression analysis for the effect of initial *V*O_2_max on running economy effect size in high load training. *ES* effect size, *J* number of effect sizes within studies

**Fig. 9:**
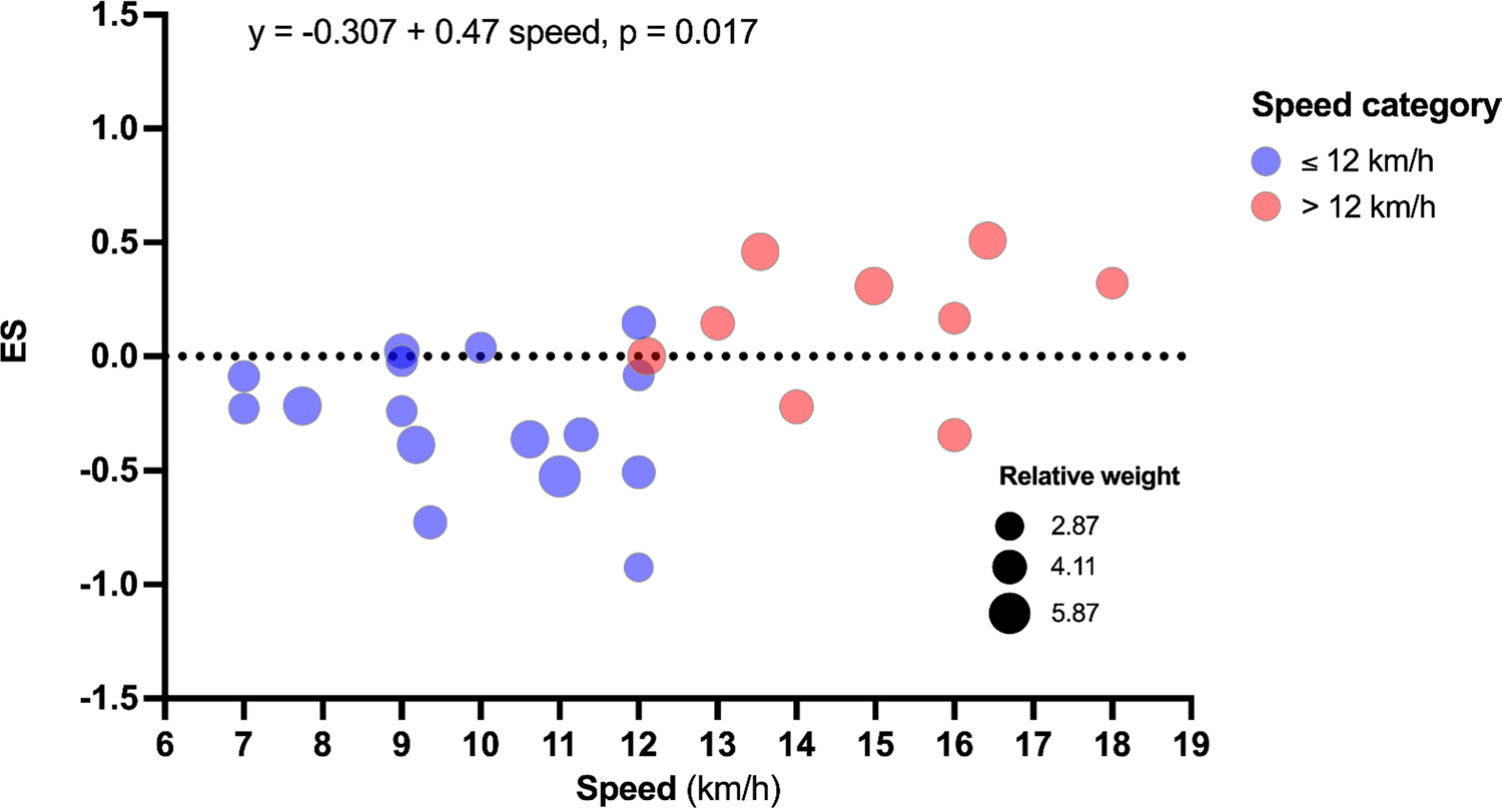
Sub-group analysis for the effect of absolute speed (categorical) on running economy effect size in plyometric training. *ES* effect size, *J* number of effect sizes within studies

A beneficial moderation for the effect of HL on RE was noted for continuous absolute speed (*β*_1_ = − 0.177, *p* = 0.027, Table 5 and Fig. 7), categorical speed (*β*_1_ = − 0.653, *p* = 0.021, Table 5) and initial (before intervention) *V*O_2_max (*β*_1_ = − 0.040, *p* = 0.020, Table 5 and Fig. 8). In PL, moderator analysis showed significant detrimental moderation in categorical speed (*β*_1_ = 0.470, *p* = 0.017, Table 6 and Fig. 9). In combined methods group, significant beneficial moderation was observed for continuous absolute speed (*β*_1_ = − 0.263, *p* = 0.003), categorical speed (*β*_1_ = − 0.679, *p* = 0.020) and U-shaped RE-speed relationship (*β*_1_ = − 0.385, *β*_2_ = − 2.008, *p* = 0.001). However, when outliers were removed, no moderating effects were observed [all *p* > 0.059, Table S3 (OSM)]. An analysis of possible moderators for SL and ISO was not performed because the minimum number of studies (i.e., eight studies) to perform the analysis was not reached.

**Table 5:** Results of meta-regression and subgroup analyses in search of possible moderators of high load training on running economy

High load training	*n* groups	*J*	*β*_0_ Hedges'* g* (SE)	95% CI	*t*_0_(*df*),* p* value	*β*_1_ Hedges' *g* (SE)	95% CI	*t*_1_(*df*),* p* value	*F*(*df*_1_, *df*_2_),* p* value
Subject characteristics									
Sex	11	19							*F*(2,16) = 1.076, *p* = 0.364
Male	6	11	− 0.360 (0.162)	− 0.703 to − 0.170	***t*(16) = **− **2.226, *p* = 0.041**				
Female	2	2	0.203 (0.349)	− 0.536 to 0.943	*t*(16) = − 1.465, *p* = 0.162	0.563 (0.384)	− 0.252 to 1.378	*t*(16) = 1.465, *p* = 0.162	
Male–female	3	6	− 0.280 (0.226)	− 0.758 to 0.198	*t*(16) = − 0.289, *p* = 0.777	0.080 (0.278)	− 0.508 to 0.668	*t*(16) = 0.289, *p* = 0.777	
Age	11	19	− 0.724 (0.519)	− 1.818 to 0.371	*t*(17) = − 1.395, *p* = 0.181	0.014 (0.016)	− 0.019 to 0.047	*t*(17) = 0.908, *p* = 0.377	*F*(1,17) = 0.824, *p* = 0.377
Body mass	11	19	− 0.973 (1.259)	− 3.629 to 1.682	*t*(17) = − 0.773, *p* = 0.450	0.010 (0.018)	− 0.028 to 0.048	*t*(17) = 0.565, *p* = 0.58	*F*(1,17) = 0.319, *p* = 0.580
Height	10	17	4.845 (5.159)	− 6.15 to 15.841	*t*(15) = 0.939, *p* = 0.362	− 0.029 (0.029)	− 0.092 to 0.033	*t*(15) = − 0.996, *p* = 0.335	*F*(1,15) = 0.992, *p* = 0.335
*V*O_2_max	10	16	1.991 (0.873)	0.117 to 3.864	***t*(14) = 2.279, *p* = 0.039**	− 0.040 (0.015)	− 0.072 to − 0.007	***t*(14) = **− **2.619, *p* = 0.02**	***F*(1,14) = 6.862, *p* = 0.020**
Performance level	11	19							*F*(2,16) = 2.837, *p* = 0.088
Moderately trained	5	8	0.018 (0.198)	− 0.401 to 0.437	*t*(16) = 0.089, *p* = 0.930				
Well trained	3	5	− 0.201 (0.187)	− 0.597 to 0.195	*t*(16) = − 1.076, *p* = 0.298	− 0.219 (0.272)	− 0.796 to 0.358	*t*(16) = − 0.804, *p* = 0.433	
Highly trained	3	6	− 0.651 (0.206)	− 1.089 to − 0.214	***t*(16) = **− **3.159, *p* = 0.006**	− 0.669 (0.286)	− 1.275 to − 0.064	***t*(16) = **− **2.343, *p* = 0.032**	
Strength training experience	7	10							*F*(1,8) = 0.795, *p* = 0.399
No	6	8	− 0.232 (0.159)	− 0.599 to 0.135	*t*(8) = − 1.459, *p* = 0.183				
Yes	1	2	0.103 (0.341)	− 0.683 to 0.890	*t*(8) = 0.303, *p* = 0.770	0.336 (0.376)	− 0.532 to 1.204	*t*(8) = 0.892, *p* = 0.399	
Strength training intervention									
Weeks	11	19	0.533 (0.487)	− 0.494 to 1.560	*t*(17) = 1.095, *p* = 0.289	− 0.093 (0.055)	− 0.21 to 0.024	*t*(17) = − 1.682, *p* = 0.111	*F*(1,17) = 2.828, *p* = 0.111
Sessions per week	11	19	− 0.214 (0.512)	− 1.295 to 0.868	*t*(17) = − 0.417, *p* = 0.682	− 0.022 (0.206)	− 0.456 to 0.415	*t*(17) = − 0.106, *p* = 0.917	*F*(1,17) = 0.011, *p* = 0.917
Total sessions	11	19	0.139 (0.406)	− 0.716 to 0.995	*t*(17) = 0.344, *p* = 0.735	− 0.020 (0.019)	− 0.060 to 0.020	*t*(17) = − 1.042, *p* = 0.312	*F*(1,17) = 1.086, *p* = 0.312
Speed assessed									
Absolute speed (continuous)	10	18	1.178 (0.588)	− 0.069 to 2.424	*t*(16) = 2.003, *p* = 0.062	− 0.117 (0.048)	− 0.219 to − 0.015	***t*(16) = **− **2.440, *p* = 0.027**	***F*(1,16) = 5.955, *p* = 0.027**
Absolute speed (category)	10	18							***F*(1,16) = 6.573, *p* = 0.021**
≤ 12.00 km/h	9	13	− 0.060 (0.130)	− 0.336 to 0.216	*t*(16) = − 0.460, *p* = 0.652				
> 12.00 km/h	3	5	− 0.713 (0.219)	− 1.177 to − 0.249	***t*(16) = **− **3.258, *p* = 0.005**	− 0.653 (0.255)	− 1.193 to − 0.113	***t*(16) = **− **2.564, *p* = 0.021**	
U-shaped RE-speed relationship (category)	10	18							*F*(2,15) = 3.552, *p* = 0.055
< 11.50 km/h	6	8	− 0.136 (0.167)	− 0.491 to 0.220	*t*(15) = − 0.814, *p* = 0.429				
11.50–14.50 km/h	5	5	0.059 (0.209)	− 0.386 to 0.504	*t*(15) = 0.282, *p* = 0.782	0.195 (0.267)	− 0.375 to 0.764	*t*(15) = 0.728, *p* = 0.478	
> 14.50 km/h	3	5	− 0.713 (0.219)	− 1.18 to − 0.247	***t*(15) = **− **3.258, *p* = 0.005**	− 0.577 (0.275)	− 1.164 to 0.009	*t*(15) = − 2.098, *p* = 0.053	
Speed relative to AT	9	16							*F*(1,14) = 0.057, *p* = 0.815
≤ AT	9	13	− 0.322 (0.156)	− 0.658 to 0.013	***t*(14) = **− **2.060, *p* = 0.059**				
> AT	3	3	− 0.403 (0.314)	− 1.077 to 0.271	*t*(14) = − 1.282, *p* = 0.221	− 0.081 (0.338)	− 0.806 to 0.645	*t*(14) = − 0.238, *p* = 0.815	
Speed relative to *V*O_2_max	10	16	0.189 (1.019)	− 1.996 to 2.374	*t*(14) = 0.186, *p* = 0.855	− 0.006 (0.013)	− 0.035 to 0.023	*t*(14) = − 0.464, *p* = 0.650	*F*(1,14) = 14.15, *p* = 0.439

In the subgroup analysis (categorical variables), the first variable of the category was considered as the reference

*AT* anaerobic threshold, *CI* confidence interval, *df* degrees of freedom, *n groups* number of experimental groups, *J* number of effect sizes, *SE* standard error

Results in bold represent a significant effect (*α* = 0.05)

**Table 6:** Results of meta-regression and subgroup analyses in search of possible moderators of plyometric training on running economy

Plyometric training	*n* groups	*J*	*β*_0_ Hedges' *g* (SE)	95% CI	*t*_0_(*df*),* p* value	*β*_1_ Hedges' *g* (SE)	95% CI	*t*_1_(*df*), *p* value	*F*(*df*_1_,* df*_2_),* p* value
Subject characteristics									
Sex	12	28							*F*(2,25) = 1.068, *p* = 0.359
Male	5	9	− 0.247 (0.157)	− 0.569 to 0.076	*t*(25) = − 1.574, *p* = 0.128				
Female	2	6	− 0.266 (0.206)	− 0.690 to 0.157	*t*(25) = − 1.295, *p* = 0.207	− 0.020 (0.258)	− 0.552 to 0.513	*t*(25) = − 0.076, *p* = 0.940	
Male–female	5	13	− 0.002 (0.119)	− 0.247 to 0.243	*t*(25) = − 0.018, *p* = 0.986	0.244 (0.197)	− 0.161 to 0.650	*t*(25) = 1.242, *p* = 0.226	
Age	12	28	− 0.016 (0.708)	− 1.472 to 1.440	*t*(26) = − 0.023, *p* = 0.982	− 0.004 (0.023)	− 0.052 to 0.044	*t*(26) = − 0.165, *p* = 0.870	*F*(1,26) = 0.027, *p* = 0.870
Body mass	12	28	3.834 (2.463)	− 1.229 to 8.897	*t*(26) = 1.557, *p* = 0.132	− 0.057 (0.036)	− 0.131 to 0.016	*t*(26) = − 1.609, *p* = 0.120	*F*(1,26) = 2.588, *p* = 0.120
Height	10	25	7.055 (5.049)	− 3.390 to 17.50	*t*(23) = 1.397, *p* = 0.176	− 0.042 (0.029)	− 0.103 to 0.019	*t*(23) = − 1.432, *p* = 0.166	*F*(1,23) = 2.052, *p* = 0.166
*V*O_2_max	12	28	− 0.289 (0.556)	− 1.433 to 0.854	*t*(26) = − 0.520, *p* = 0.607	0.003 (0.011)	− 0.019 to 0.026	*t*(26) = 0.291, *p* = 0.773	*F*(1,26) = 0.085, *p* = 0.773
Performance level	12	28							
Moderately trained	5	16	− 0.06 (0.114)	− 0.294 to 0.174	*t*(25) = − 0.528, *p* = 0.602				*F*(1,25) = 1.342, *p* = 0.280
Well trained	6	10	− 0.283 (0.143)	− 0.577 to 0.110	*t*(25) = − 1.982, *p* = 0.059	− 0.223 (0.182)	− 0.598 to 0.153	*t*(25) = − 1.222, *p* = 0.233	
Highly trained	1	2	0.244 (0.346)	− 0.469 to 0.956	*t*(25) = 0.704, *p* = 0.488	0.304 (0.364)	− 0.446 to 1.054	*t*(25) = 0.834, *p* = 0.412	
Strength training experience	8	22							*F*(1,20) = 2.176, *p* = 0.156
No	6	18	− 0.221 (0.122)	− 0.476 to 0.033	*t*(20) = − 1.815, *p* = 0.085				
Yes	2	4	0.192 (0.252)	− 0.334 to 0.718	*t*(20) = 0.761, *p* = 0.455	0.413 (0.280)	− 0.171 to 0.998	*t*(20) = 1.475, *p* = 0.156	
Strength training intervention									
Weeks	12	28	− 0.158 (0.373)	− 0.925 to 0.608	*t*(26) = − 0.425, *p* = 0.674	0.004 (0.048)	− 0.096 to 0.103	*t*(26) = 0.076, *p* = 0.940	*F*(1,26) = 0.006, *p* = 0.940
Sessions per week	12	28	− 0.152 (0.344)	− 0.858 to 0.554	*t*(26) = − 0.442, *p* = 0.662	0.011 (0.156)	− 0.310 to 0.331	*t*(26) = 0.069, *p* = 0.945	*F*(1,26) = 0.005, *p* = 0.945
Total sessions	12	28	− 0.238 (0.354)	− 0.966 to 0.490	*t*(26) = − 0.672, *p* = 0.507	0.007 (0.022)	− 0.039 to 0.053	*t*(26) = 0.320, *p* = 0.751	*F*(1,26) = 0.103, *p* = 0.751
Speed assessed									
Absolute speed (continuous)	11	25	− 0.755 (0.378)	− 1.538 to 0.028	*t*(23) = − 1.995, *p* = 0.058	0.055 (0.031)	− 0.010 to 0.119	*t*(23) = 1.737, *p* = 0.096	*F*(1,23) = 3.016, *p* = 0.096
Absolute speed (category)	11	27							***F*(1,25) = 6.526, *p* = 0.017**
≤ 12.00 km/h	10	18	− 0.307 (0.110)	− 0.532 to 0.081	***t*(25) = **− **2.802, *p* = 0.010**				
> 12.00 km/h	5	9	0.163 (0.148)	− 0.141 to 0.468	*t*(25) = 1.104, *p* = 0.280	0.470 (0.184)	0.091 to 0.849	***t*(25) = 2.555, *p* = 0.017**	
U-shaped RE-speed relationship (category)	11	27							*F*(1,24) = 2.187, *p* = 0.134
< 11.50 km/h	8	15	− 0.276 (0.118)	− 0.521 to − 0.032	***t*(24) = **− **2.335, *p* = 0.028**				
11.50–14.50 km/h	5	7	− 0.108 (0.174)	− 0.467 to 0.251	*t*(24) = − 0.620, *p* = 0.541	0.169 (0.210)	− 0.265 to 0.603	*t*(24) = 0.802, *p* = 0.431	
> 14.50 km/h	3	5	0.210 (0.202)	− 0.206 to 0.626	*t*(24) = 1.042, *p* = 0.308	0.486 (0.234)	0.004 to 0.969	***t*(24) = 2.080, *p* = 0.048**	
Speed relative to AT	8	22							*F*(1,20) = 2.722, *p* = 0.115
≤ AT	8	16	− 0.246 (0.114)	− 0.484 to 0.008	***t*(20) = **− **2.155, *p* = 0.044**				
> AT	4	6	0.109 (0.183)	− 0.804 to 0.094	*t*(20) = − 1.650, *p* = 0.115	0.355 (0.215)	− 0.094 to 0.804	*t*(20) = 1.650, *p* = 0.115	
Speed relative to *V*O_2_max	9	18	− 0.349 (0.741)	− 1.920 to 1.222	*t*(16) = − 0.471, *p* = 0.644	0.001 (0.010)	− 0.020 to 0.023	*t*(16) = 0.144, *p* = 0.887	*F*(1,16) = 0.021, *p* = 0.887

In the subgroup analysis (categorical variables), the first variable of the category was considered as the reference

*AT* anaerobic threshold, *CI* confidence interval, *df* degrees of freedom, *n groups* number of experimental groups, *J* number of effect sizes, *SE* standard error

Results in bold represent a significant effect (*α* = 0.05)

## Discussion

The purpose of this systematic review with meta-analysis was to evaluate the effect of different formats of strength training methods (i.e., HL, SL, PL, ISO, and combined methods) on RE in middle- and long-distance runners and to examine the effect of strength training on RE as a function of assessed running speed. The main findings indicate that HL and combined methods formats induced a small improvement in RE, whereas no significant main effect was found after SL or ISO. Moderator analyses revealed that when HL was adjusted for absolute categorical speed (i.e., ≤ 12.00 km/h and > 12.00 km/h) or absolute continuous speed (i.e., 8.64 km/h to 17.85 km/h), or *V*O_2_max, the higher the speed or *V*O_2_max, the greater the beneficial effect on RE. In contrast, when PL was adjusted for absolute categorical speed, it induced an improvement in RE at speeds ≤ 12.00 km/h. These results suggest that HL, PL, and combined methods can improve RE, although the beneficial effect can be moderated by factors such as RE speed selected for assessment and athletes’ fitness level (i.e., *V*O_2_max levels). These results and their implications are discussed in the sections below.

### Main Analysis

#### High Load Training

HL is characterised by low-speed exercises and high force requirements (≥ 80% 1 RM or ≤ 7 RM) which aim to improve the development of maximal strength [15]. The main analysis revealed that HL induced a small improvement on RE (ES = − 0.266, *p* = 0.039), a result that is in line with other meta-analyses on this topic [4, 5, 24, 120]. These improvements were observed in interventions of between 6 and 14 weeks’ duration with a training frequency of 2–4 days per week. There appeared to be no moderating effect of duration in weeks, sessions per week or total sessions on RE (all *p* > 0.111). These results are contrary to those of a recent meta-analysis [120] which found that the implementation of HL over a period of ten weeks or more had a greater effect on RE compared to shorter programmes. This is possibly because the authors of that study included ISO in the analysis, adding two further studies [116, 121] that incorporated 14-week programmes. In contrast, in the current analysis, just one study [103] included a training programme of 14 weeks’ duration. However, despite finding no significant moderating effect of training duration, the slope of the curve in the conducted meta-regression was negative (i.e., the longer the duration of the study, the better the RE; *β*_1_ = − 0.09, *p* = 0.111). It is therefore possible that studies of longer intervention duration may induce an effect of HL on RE.

The improvement of RE following HL may be due to different mechanisms. It is known that HL may induce neuromuscular changes, such as altered recruitment and firing frequency of motor units and changes in fibre type, resulting in increased rate of force development (RFD) [99, 122]. The early-phase RFD (e.g., isometric mid-thigh pull; 90° squat) has been correlated with RE at 10.00 km/h [123], 12.00 km/h [124], and speed corresponding at 70% *V*O_2_max [99]. For example, in a study by Støren et al. [99] it was found that eight weeks of HL improved RFD by 26% in the 90° squat and that this correlated with improvements in RE pre- and post-intervention. Furthermore, this increase occurred independent of changes in body weight, and thus the reported increase in RFD may have been due to neuromuscular adaptations [122]. A greater RFD after HL interventions would allow athletes to generate higher levels of force in short periods of time, allowing a rapid transition from the braking phase to the propulsion phase of the gait cycle, promoting favourable muscular conditions [123] that maximise the force–velocity relationship [125] and, thus, RE. On the other hand, HL may improve RE due to changes in lower limb stiffness [123, 126, 127], which would result in more efficient energy storage and release from the lower limbs, thus reducing the energy cost of running [128]. For example, a study by Millet et al. [103] reported significant increases in RE after 14 weeks of HL and this was accompanied by an increase in leg stiffness.

#### Submaximal Load Training

Only three studies included SL (Fig. 4). The main analysis found no significant effect of this strength training method on RE at speeds between 9.75 to 16.00 km/h (*p* = 0.131), which could be attributed to several reasons. It has been found that SL training is not as intense a stimulus as HL for the generation of adaptations in muscle–tendon stiffness [129]. For instance, in a study by Piacentini et al. [19], a significant improvement in RE was found after HL whereas no significant improvement was observed after SL. On the other hand, it seems that SL is not as effective a stimulus for improving stretch-shortening cycle function as PL [130]. For example, Berryman et al. [114] found that both PL and SL improved RE but the percentage improvement was greater after PL (7% vs 4%). In another study [115], PL combined with SL and isolated SL improved RE at 12.00 km/h, whilst improvement in RE at 16.00 km/h was only found in PL combined with SL and not SL as a singular training method.

However, these results should be interpreted with caution, as only three studies were included in the analysis meaning that the conclusion could be undermined by low statistical power. Moreover, an analysis of possible moderators was not possible in this case.

#### Plyometric Training

The main analysis found no significant effect of PL on RE (*p* = 0.167). This result does not align with recent systematic reviews with meta-analysis related to the effect of strength training on RE in endurance runners [5, 43, 120]. For example, it has been suggested that PL may have a greater effect in athletes with higher performance levels [43, 120] or in training programmes of longer duration in endurance athletes [120] and healthy adults (i.e., longer than 7 weeks) [131]. However, we found no moderating effect of participant characteristics or intervention duration (all *p* > 0.120, Table 6). From the studies included in the analysis, improvements in RE were found in moderately trained athletes [12] and in 6-week intervention programmes [12, 106, 107]. The lack of any significant effect of PL in the main analysis is possibly due to methodological differences with other meta-analyses that have been carried out on this topic. For example, two meta-analyses [5, 43] included PL with resistance training within the same analysis while only one meta-analysis [120] included isolated PL interventions, as in the current study. This may be relevant given that PL combined with other strength training methods may have a greater effect on RE (see Sect. 4.1.5). Furthermore, the difference in the speeds used to evaluate RE could also have given rise to the discrepancies observed between various different studies [9]. Contrary to the results of other meta-analyses [5, 43, 120], we included all effect sizes that were documented within each study (i.e., different speeds at which RE was assessed). Interestingly, after performing sub-group analysis, we found that PL had a beneficial effect when the speeds are less or equal to 12.00 km/h compared to when the speed is higher than 12.00 km/h (*β*_1_ = 0.47, *p* = 0.017, Fig. 9). Therefore, from these results it is possible that the improvement in RE is primarily influenced by running speed, rather than performance level and/or duration of training programmes.

#### Isometric Training

ISO is characterised by exercises that require muscle contraction without external movement. ISO can improve RFD [17] and tendon stiffness (i.e., Achilles tendon) [116, 117] without changes in joint stiffness [132, 133], adaptations that could be related to improved RE [117, 123]. However, the main analysis found no significant effect of ISO on RE (*p* = 0.253). A possible explanation for this result may be due to the difference in muscle action times that were evaluated in the various different studies. For example, of the three studies [112, 116, 117] included in this analysis, just one [117] showed no improvement in RE. Although all three studies performed the same exercise (i.e., isometric ankle plantarflexion) at intensities equal to or greater than 80% of the maximal voluntary contraction, they differed in muscle action times. While two studies [112, 116] used muscle action times of 3 s, in the study by Fletcher et al. [117] the action time was 20 s. This could be relevant because it is known that improvement in RFD is determined by neuromuscular adaptations, muscle size and tendon stiffness [134] and isometric efforts of 1–5 s have been suggested to generate such neuromuscular adaptations [17]. Given that improvements in tendon stiffness were found in both short duration (i.e., 3 s) [116] and long duration (i.e., 20 s) [117] isometric efforts, it is possible that the failure to improve RE was due to an inadequate stimulus to the neuromuscular system in the way that short duration efforts at maximal speed do. However, these interpretations need to be made with caution given the small number of studies in the analysis and the small sample sizes of those studies. In addition, in the three included studies [112, 116, 117], participants performed the same single-joint exercise (i.e., ankle plantarflexion), while the other strength training methods included multi-joint exercises (e.g., squat or jump squat). Therefore, additional investigations are needed to elucidate the effects of ISO on RE including multi-joint exercises (e.g., specific hip or knee run exercises).

#### Combined Methods Training

Various different studies implemented more than one strength training method, such as SL with PL [8, 102, 115, 118, 119], HL with SL [135, 136] and HL with PL [20]. From the main analysis, we found that combined methods group had a significant small effect on RE (ES = − 0.426, *p* = 0.018), which was superior to that found in HL and PL after adjusting for categorical speed. One possible explanation for this is that all studies included in the analysis included PL and/or HL. Therefore, it could be hypothesised that the different adaptations induced by these strength training methods could complement each other when included in the same programme, generating a greater effect on RE [20]. However, it is important to mention that most studies included SL [102, 115, 118, 119, 135–137] and given that in the individual analysis of this strength training method we found no significant effect on RE (see Sect. 4.1.2), it is possible that this type training, executed in isolation, may not be enough of a stimulus to generate changes in RE. However, combined with other types of strength training it may be [20, 114, 115].

On the other hand, these strength training methods used were either combined within the same training session [20, 102, 115, 118, 119, 137] or performed in a different part of the programme [135, 136]. In the first case, we found different types of exercise sequences within training sessions, such as traditional and complex training (Table 2). A recent meta-analysis found that different exercise sequences can improve the force- and velocity-producing capabilities of an athlete [18]. For example, a complex sequence can be used combining heavy exercises (e.g., HL and/or SL exercises) followed by light exercises (e.g., PL and/or SL exercises), thus inducing post-activation performance enhancement by improving the speed at which PL exercises are executed [18]. This concept refers to the phenomenon in which maximal strength, power and speed are increased after a conditioned contraction [138]. Traditional training employs light exercises followed by heavy exercises which can enhance strength development [18]. The moderator analysis of exercise sequence in this meta-analysis showed no significant moderating effect on this variable (*p* = 0.956). This may be because the strength training method is more influential than the sequence of exercises within a prescribed training session. Also, it is important to mention that complex training also has different sequences within it (e.g., ascending, descending, French contrast and contrast) that can generate different adaptations [139]. Therefore, future research could investigate the effect of different strength training methods and with different complex training strategies on RE.

### Strength Training and Running Economy Speed

#### Absolute Speed (Continuous and Categorical)

Absolute and categorical speed acted as beneficial moderators on the effect of HL on RE (*β*_1_ = − 0.177, *p* = 0.027, Fig. 7; *β*_1_ = − 0.653, *p* = 0.021, respectively). Since the increase in energy cost as speed increases could be the result of an increase in muscle energy cost to generate higher levels of force in short periods of time [125], an increase in RFD may be reflected particularly at higher running speeds. Additionally, we found a significant moderator effect of *V*O_2_max on RE in HL (*β*_1_ = − 0.05, *p* = 0.02, Fig. 8). Indeed, it has been observed that the correlation between leg stiffness and RE increases with *V*O_2_max [127]. Given that more highly trained athletes make more efficient use of elastic energy (i.e., the Achilles tendon) to minimise muscle energy cost [140], coupled with a possible increase in tendon stiffness generated by HL [133], it is possible that athletes with higher levels of performance (i.e., higher initial *V*O_2_max) may be better able to transfer these adaptations to running at a lower energy cost. However, it is possible that the speeds chosen to assess RE were in line with the performance level of the runners, with lower speeds for lower-level runners and higher speeds for higher level runners.

Aside from HL, we found that in PL categorical speed has a positive moderating (i.e., detrimental) effect on RE (*β*_1_ = 0.47, *p* = 0.017, Fig. 9). It has been observed that PL can improve joint stiffness in runners [107] and healthy individuals [141], which may be due to an increase in tendon elongation (i.e., Achilles tendon) and a decrease in fascicle length (i.e., medial gastrocnemius) [141]. A more compliant tendon could store and release more elastic energy, decreasing muscle energy cost, in situations where substantial pre-stretching occurs [125], as at low running speeds. Conversely, it has been found that in plantar flexors, as speed increases, tendon energy storage and release become prioritised over muscle work [142], and thus a more compliant tendon could be detrimental. In fact, in the study by Pellegrino et al. [12] it was found that after 6 weeks of PL, RE improved at speeds ranging from 7.74 to 10.62 km/h, while no improvement or detriment was observed at speeds between 12.10 and 16.42 km/h. Taken together, it appears that HL and PL may improve RE but with varied effects depending on running speed. However, future research is required to elucidate the possible mechanisms of RE improvement.

#### U-Shaped RE–Speed Relationship

Several studies have found a U-shaped relationship between the energy cost of running and speed (from 8.00 to 18.00 km/h) [23, 143], with elastic energy being independent of running speed [23]. This higher energy cost at low speeds (i.e., < 11.50 km/h) may be due to greater muscle activation for greater neuromotor control [144], whereas at high speeds (i.e., > 14.50 km/h) it may be due to the muscle being in a less favourable contractile condition as a priority for storage and release of elastic energy from the tendon [142]. When we performed the moderator analysis with the variable U-shaped RE–speed relationship, we did not find a significant moderating effect for HL, PL or combined methods. However, we did find a near-significant moderating effect for HL (*p* = 0.055). Indeed, the almost significant regression coefficient showed a beneficial effect at higher speed (*β*_2_ = − 0.557, *p* = 0.053) compared to the regression coefficient at moderate speed (*β*_1_ = − 0.195, *p* = 0.478). This could suggest a role of HL in improving RE at high speeds, covering the higher muscle energy cost at high speeds. A possible explanation for finding a moderating effect on absolute speed and not on U-shaped RE–speed relationship may be due to the wide range of athletes that were included in this analysis. It was reported that recreational athletes had a curvilinear energy cost relationship at a range of speeds lower than those observed in highly trained athletes [143]. Therefore, future research could analyse the impact of HL on RE at higher speeds where muscle energy cost is higher, as well as consider the difference between athletes of different performance levels.

#### Speed Relative to Anaerobic Threshold

The assessment of RE at speeds relative to the anaerobic threshold has been suggested [22] as this allows consideration of the differences in energy substrates and anaerobic threshold [21] or running at a race pace (e.g., at marathon pace), with the intention of equalising the metabolic conditions of the runners [22]. For example, Piacentini et al. [19] found that HL improved RE only at marathon running pace speed, whereas at 1.00 km/h below or above marathon pace no improvement was found. On the other hand, when the speed is above the anaerobic threshold, anaerobic metabolism starts to become relevant, and it is recommended to correct the values by adding blood lactate energy values [30, 145]. When anaerobic metabolism is added to the energy cost, it has a linear relationship with running speed [30, 145]. However, among the studies that included groups with values above the anaerobic threshold, only one study [113] reported blood lactate concentration values, and thus only one study would allow the application of correction procedures to adjust for anaerobic metabolism contribution. Surprisingly, this study [113] found that PL improved RE at 18.00 km/h (measured in LO_2_/min); however, a detrimental effect was noted after adjusting for blood lactate values. In the moderator analysis performed with the categorical speed relative to anaerobic threshold we did not find a moderating effect for this variable (all *p* > 0.115), possibly because the number of groups with speeds less than or equal to the anaerobic threshold was considerably higher than the number of groups with speeds greater than the anaerobic threshold. It is therefore recommended that future research should include the contribution of anaerobic metabolism when assessing RE at speeds above the anaerobic threshold, allowing the effect of strength training on RE at higher speeds to be assessed.

#### Speed Relative to *V*O_2_max

It has been found that athletes are more economical at the speeds at which they compete (i.e., at middle- or long-distance speeds) and that differences in RE between men and women are not significant when assessed at relative running intensity (i.e., as a percentage of *V*O_2_max) [146]. Therefore, we created a new variable whereby speeds were estimated as speed relative to *V*O_2_max. However, we found no moderating effect of this variable in HL, PL, or combined methods (all *p* > 0.419). This may be because only two studies [99, 103] assessed running economy at speeds relative to *V*O_2_max, while the other values were estimated. On the other hand, it is possible that speed relative to *V*O_2_max may not consider differences in energy substrates as speed relative to anaerobic threshold would.

### Strengths and Limitations

Some limitations of this meta-analysis should be mentioned. Firstly, we performed analyses separately for each strength training method due to their different compositions and this limited the number of studies (i.e., < 8) for which a moderator analysis for the effects of SL and ISO could be performed. Secondly, in terms of speeds assessed in RE, all but six studies [99, 100, 103, 111, 117, 118] used absolute speeds; however this does not consider the difference in energy substrates and anaerobic threshold [21], so it is recommended to use speeds relative to anaerobic threshold or relative to race pace [22]. The strengths of this meta-analysis should also be acknowledged. To our knowledge, this is the first meta-analysis to investigate the moderation of assessed speed on the effect of strength training on RE by including all assessed speeds from each study, allowing the effect of different strength training methods on RE at different running speeds to be elucidated.

## Conclusions

Based on these results, HL, PL, and combined methods can improve RE. Furthermore, PL improves RE at speeds of ≤ 12.00 km/h, combined methods group at 10.00 to 14.45 km/h and, HL at 8.64 to 17.85 km/h (particularly at higher speeds), and as a function of athletes *V*O_2_max level. No RE improvement was noted after SL or ISO. Therefore, athletes and coaches might consider including different strength training methods (HL, PL and/or combined methods) in traditional endurance training to improve running economy at different speed ranges in middle- and long-distance runners. Future experimental research is needed to understand the potential effects, and underlying mechanisms, of different strength training methods on RE assessed at different speeds in middle- and long-distance runners, particularly among under-researched populations (e.g., females; highly trained athletes).

### Supplementary Information

Below is the link to the electronic supplementary material.

Supplementary file1 (DOCX 2775 KB)

## Declarations

### Data Availability

All data generated or analysed during this systematic review and meta-analysis are included in the article as table(s), figure(s), and/or Online Supplementary Material(s). Any other data requirement can be directed to the corresponding author upon reasonable request.
