# Supplementary material for: Effect of Strength Training Programs in Middle- and Long-Distance Runners’ Economy at Different Running Speeds: A Systematic Review with Meta-analysis
Source: Sports Med. 2024 Jan 2;54(4):895–932. doi: 10.1007/s40279-023-01978-y (PMC11052887; doi:10.1007/s40279-023-01978-y)
Supplement: Supplementary file 1 — Supplementary file1 (DOCX 2775 KB) [file 40279_2023_1978_MOESM1_ESM.docx]

**Effect of strength training programs in middle- and long-distance runners’ economy at different running speeds: A systematic review with meta-analysis**

**Short title:** Strength training and running economy.

Cristian Llanos-Lagos ^1^, Rodrigo Ramirez-Campillo ^2^, Jason Moran ^3^, Eduardo Saez de Villarreal ^1^

^1^ Physical Performance Sports Research Center (PPSRC), Universidad Pablo de Olavide, 41704 Sevilla, Spain.

^2^ Exercise and Rehabilitation Sciences Institute. School of Physical Therapy. Faculty of Rehabilitation Sciences. Universidad Andres Bello. Santiago 7591538, Chile.

^3^ School of Sport, Rehabilitation and Exercise Sciences, University of Essex, Colchester, Essex, CO43SQ, UK.

**Corresponding author**

MSc. Cristian Llanos-Lagos

Universidad Pablo de Olavide

Physical Performance Sports Research Center

41704 Sevilla

Spain

Email: cfllalag@alu.upo.es

ORCID: 0009-0003-6906-4977

**Electronic Supplementary Material Table S1**

**Article tittle:** Effect of strength training programs in middle- and long-distance runners’ economy at different running speeds: A systematic review with meta-analysis

**Author names:** Cristian Llanos-Lagos, Rodrigo Ramirez-Campillo, Jason Moran, Eduardo Saez de Villarreal

**Affiliation and e-mail of the corresponding author:**

MSc. Cristian Llanos-Lagos

Universidad Pablo de Olavide

Physical Performance Sports Research Center

41704 Sevilla

Spain

Email: cfllalag@alu.upo.es

ORCID: 0009-0003-6906-4977

**Table S1.** Search strategy

| **Database** | **Full search strategy** |
| --- | --- |
| **PubMed** | (("Marathon Running"[Mesh] OR “Running”[Mesh] OR “Distance run*”[Title] OR “run”[Title] OR “Running” [Title] OR “Middle distance*”[Title] OR “Long distance*”[Title] OR “Endurance athlete*” [Title] OR “800 m*” [Title] OR “1500 m*” [Title] OR “3000 m”[Title] OR “5000 m” [Title] OR “5 km” [Title] OR “5km” [Title] OR “10km” [Title] OR “10 km” [Title] OR “Half marathon*”[Title] OR “Cross countr*”[Title] OR “Marathon*”[Title]) AND (“Resistance Training”[Mesh] OR "Plyometric Exercise"[Mesh] OR "Weight Lifting"[Mesh] OR “Strength training”[Title] OR “Weight training” [Title] OR “Resistance training” [Title] OR “Resistance exercise*”[Title] OR “Concurrent training”[Title] OR “Hypertrophy”[Title] OR “Exercise program*”[Title] OR “Weight-bearing” [Title] OR “Muscular strength”[Title] OR “Muscle strength”[Title] OR “Isoinertial”[Title] OR “Plyometric”[Title] OR “Stretch shortening exercise*”[Title] OR “Shortening contraction”[Title] OR “Reactive strength”[Title] OR “Power training”[Title] OR “Weightlifting”[Title] OR “Weight lifting”[Title] OR “Explosive strength”[Title] OR “Ballistic”[Title] OR “Local muscular”[Title] OR “TRX suspension training”[Title] OR “Suspension training”[Title] OR “Free weight training”[Title] OR “Strengthening”[Title] OR “Flywheel”[Title] OR “Eccentric training”[Title] OR “Eccentric contraction”[Title] OR “Eccentric exercis*”[Title] OR “Eccentric-weight”[Title] OR “Eccentric load*”[Title] OR “Negative muscle work”[Title])) AND (("Oxygen Consumption"[Mesh] OR "Adaptation, Physiological"[Mesh] OR “Maximal oxygen*” OR “Maximum oxygen*” OR “Peak oxygen*” OR “VO2max” OR “VO2peak” OR “Aerobic power” OR “Aerobic capacity” OR “Aerobic endurance” OR “Endurance performance” OR “Cardiovascular performance” OR “Cardiorespiratory fitness”) AND/OR (“vVO2max” OR “Velocity at maximal oxygen*” OR “Maximal aerobic velocity” OR “Maximum aerobic velocity” OR “Maximal aerobic speed” OR “Maximum aerobic speed”) AND/OR (“Energy cost” OR “Energetic cost” OR “Caloric cost” OR “Metabolic cost” OR “Mechanical efficiency” OR “Energy cost of running” OR “Running efficiency” OR “Running economy”) AND/OR ("Anaerobic Threshold"[Mesh] OR “Anaerobic threshold” OR “Ventilatory threshold” OR “Gas exchange threshold” OR “Lactate threshold” OR “Lactate” OR “Lactate turnpoint” OR “Lactate minimum speed” OR “Maximal lactate steady state” OR “Onset of blood lactate accumulation” OR “Respiratory compensation point” OR “Critical speed”) AND/OR (“Maximal anaerobic capacity” OR “Anaerobic” OR “Anaerobic power” OR “Anaerobic capacity” OR “Anaerobic speed” OR “Maximal speed”) AND/OR (“Physical endurance”[Mesh] OR “Performance” OR “time trial” OR “Running performance” OR “Distance run*” OR “Time” OR “Running speed” OR “Running time”)) |
| **Web of Sciences (all databases)** | ((TI=(“Distance run*” OR “Run*” OR “Running” OR “Middle distance*” OR “Long distance*” OR “Endurance athlete*” OR “800 m*” OR “1500 m*” OR “3000 m” OR “5000 m” OR “5 km” OR “5km” OR “10km” OR “10 km” OR “Half marathon*” OR “Cross countr*” OR “Marathon*”)) AND TI=(“Strength training” OR “Weight training” OR “Resistance training” OR “Resistance exercise*” OR “Concurrent training” OR “Hypertrophy” OR “Exercise program*” OR “Weight-bearing” OR “Muscular strength” OR “Muscle strength” OR “Isoinertial” OR “Plyometric” OR “Stretch shortening exercise*” OR “Shortening contraction” OR “Reactive strength” OR “Power training” OR “Weightlifting” OR “Weight lifting” OR “Explosive strength” OR “Ballistic” OR “Local muscular” OR “TRX suspension training” OR “Suspension training” OR “Free weight training” OR “Strengthening” OR “Flywheel” OR “Eccentric training” OR “Eccentric contraction” OR “Eccentric exercis*” OR “Eccentric-weight” OR “Eccentric load*” OR “Negative muscle work”)) AND TS=(“Maximal oxygen*” OR “Maximum oxygen*” OR “Peak oxygen*” OR “VO2max” OR “VO2peak” OR “Aerobic power” OR “Aerobic capacity” OR “Aerobic endurance” OR “Endurance performance” OR “Cardiovascular performance” OR “Cardiorespiratory fitness” OR “vVO2max” OR “Velocity at maximal oxygen*” OR “Maximal aerobic velocity” OR “Maximum aerobic velocity” OR “Maximal aerobic speed” OR “Maximum aerobic speed” OR “Energy cost” OR “Energetic cost” OR “Caloric cost” OR “Metabolic cost” OR “Mechanical efficiency” OR “Energy cost of running” OR “Running efficiency” OR “Running economy” OR “Anaerobic threshold” OR “Ventilatory threshold” OR “Gas exchange threshold” OR “Lactate threshold” OR “Lactate” OR “Lactate turnpoint” OR “Lactate minimum speed” OR “Maximal lactate steady state” OR “Onset of blood lactate accumulation” OR “Respiratory compensation point” OR “Critical speed” OR “Maximal anaerobic capacity” OR “Anaerobic” OR “Anaerobic power” OR “Anaerobic capacity” OR “Anaerobic speed” OR “Maximal speed” OR “Performance” OR “Time trial” OR “Running performance” OR “Distance run*” OR “Time” OR “Running speed” OR “Running time”) |
| **Scopus** | TITLE (“Distance run*” OR “Run*” OR “Running” OR “Middle distance*” OR “Long distance*” OR “Endurance athlete*” OR “800 m*” OR “1500 m*” OR “3000 m” OR “5000 m” OR “5 km” OR “5km” OR “10km” OR “10 km” OR “Half marathon*” OR “Cross countr*” OR “Marathon*”) AND TITLE (“Strength training” OR “Weight training” OR “Resistance training” OR “Resistance exercise*” OR “Concurrent training” OR “Hypertrophy” OR “Exercise program*” OR “Weight-bearing” OR “Muscular strength” OR “Muscle strength” OR “Isoinertial” OR “Plyometric” OR “Stretch shortening exercise*” OR “Shortening contraction” OR “Reactive strength” OR “Power training” OR “Weightlifting” OR “Weight lifting” OR “Explosive strength” OR “Ballistic” OR “Local muscular” OR “TRX suspension training” OR “Suspension training” OR “Free weight training” OR “Strengthening” OR “Flywheel” OR “Eccentric training” OR “Eccentric contraction” OR “Eccentric exercis*” OR “Eccentric-weight” OR “Eccentric load*” OR “Negative muscle work”) AND ALL (“Maximal oxygen*” OR “Maximum oxygen*” OR “Peak oxygen*” OR “VO2max” OR “VO2peak” OR “Aerobic power” OR “Aerobic capacity” OR “Aerobic endurance” OR “Endurance performance” OR “Cardiovascular performance” OR “Cardiorespiratory fitness” OR “vVO2max” OR “Velocity at maximal oxygen*” OR “Maximal aerobic velocity” OR “Maximum aerobic velocity” OR “Maximal aerobic speed” OR “Maximum aerobic speed” OR “Energy cost” OR “Energetic cost” OR “Caloric cost” OR “Metabolic cost” OR “Mechanical efficiency” OR “Energy cost of running” OR “Running efficiency” OR “Running economy” OR “Anaerobic threshold” OR “Ventilatory threshold” OR “Gas exchange threshold” OR “Lactate threshold” OR “Lactate” OR “Lactate turnpoint” OR “Lactate minimum speed” OR “Maximal lactate steady state” OR “Onset of blood lactate accumulation” OR “Respiratory compensation point” OR “Critical speed” OR “Maximal anaerobic capacity” OR “Anaerobic” OR “Anaerobic power” OR “Anaerobic capacity” OR “Anaerobic speed” OR “Maximal speed” OR “Performance” OR “Time trial” OR “Running performance” OR “Distance run*” OR “Time” OR “Running speed” OR “Running time”) |
| **SportDiscus** | ( TI (“Distance run*” OR “Run*” OR “Running” OR “Middle distance*” OR “Long distance*” OR “Endurance athlete*” OR “800 m*” OR “1500 m*” OR “3000 m” OR “5000 m” OR “5 km” OR “5km” OR “10km” OR “10 km” OR “Half marathon*” OR “Cross countr*” OR “Marathon*”) ) AND ( TI (“Strength training” OR “Weight training” OR “Resistance training” OR “Resistance exercise*” OR “Concurrent training” OR “Hypertrophy” OR “Exercise program*” OR “Weight-bearing” OR “Muscular strength” OR “Muscle strength” OR “Isoinertial” OR “Plyometric” OR “Stretch shortening exercise*” OR “Shortening contraction” OR “Reactive strength” OR “Power training” OR “Weightlifting” OR “Weight lifting” OR “Explosive strength” OR “Ballistic” OR “Local muscular” OR “TRX suspension training” OR “Suspension training” OR “Free weight training” OR “Strengthening” OR “Flywheel” OR “Eccentric training” OR “Eccentric contraction” OR “Eccentric exercis*” OR “Eccentric-weight” OR “Eccentric load*” OR “Negative muscle work”) ) AND TX ( (“Maximal oxygen*” OR “Maximum oxygen*” OR “Peak oxygen*” OR “VO2max” OR “VO2peak” OR “Aerobic power” OR “Aerobic capacity” OR “Aerobic endurance” OR “Endurance performance” OR “Cardiovascular performance” OR “Cardiorespiratory fitness” OR “vVO2max” OR “Velocity at maximal oxygen*” OR “Maximal aerobic velocity” OR “Maximum aerobic velocity” OR “Maximal aerobic speed” OR “Maximum aerobic speed” OR “Energy cost” OR “Energetic cost” OR “Caloric cost” OR “Metabolic cost” OR “Mechanical efficiency” OR “Energy cost of running” OR “Running efficiency” OR “Running economy” OR “Anaerobic threshold” OR “Ventilatory threshold” OR “Gas exchange threshold” OR “Lactate threshold” OR “Lactate” OR “Lactate turnpoint” OR “Lactate minimum speed” OR “Maximal lactate steady state” OR “Onset of blood lactate accumulation” OR “Respiratory compensation point” OR “Critical speed” OR “Maximal anaerobic capacity” OR “Anaerobic” OR “Anaerobic power” OR “Anaerobic capacity” OR “Anaerobic speed” OR “Maximal speed” OR “Performance” OR “Time trial” OR “Running performance” OR “Distance run*” OR “Time” OR “Running speed” OR “Running time”) ) |

Mesh: Medical Subject Headings; TI: title; TS: topic; TX: full text.

**Electronic Supplementary Material Table S2**

**Article tittle:** Effect of strength training programs in middle- and long-distance runners’ economy at different running speeds: A systematic review with meta-analysis

**Author names:** Cristian Llanos-Lagos, Rodrigo Ramirez-Campillo, Jason Moran, Eduardo Saez de Villarreal

**Affiliation and e-mail of the corresponding author:**

MSc. Cristian Llanos-Lagos

Universidad Pablo de Olavide

Physical Performance Sports Research Center

41704 Sevilla

Spain

Email: cfllalag@alu.upo.es

ORCID: 0009-0003-6906-4977

**Table S2.** Physiotherapy Evidence Database (PEDro) scale.

| **Authors, year** | **N1** | **N2** | **N3** | **N4** | **N5** | **N6** | **N7** | **N8** | **Total** | **Risk of bias** |
| --- | --- | --- | --- | --- | --- | --- | --- | --- | --- | --- |
| Ache-Dias *et al.* [110] | Yes | 1 |  | 1 | 1 | 1 | 1 | 1 | 6 | Low risk |
| Albracht & Arampatzis [116] | Yes |  | 1 |  | 1 | 1 | 1 | 1 | 5 | Moderate risk |
| Berryman *et al.,* 2010 [114] | Yes | 1 |  | 1 | 1 | 1 | 1 | 1 | 6 | Low risk |
| Blagrove *et al.,* 2018 [118] | Yes | 1 |  | 1 |  | 1 | 1 | 1 | 5 | Moderate risk |
| Damasceno *et al.,* 2015 [101] | Yes | 1 | 1 | 1 | 1 | 1 | 1 | 1 | 7 | Low risk |
| do Carmo *et al.,* 2021 [108] | Yes | 1 |  | 1 | 1 | 1 | 1 | 1 | 6 | Low risk |
| Ferrauti *et al.,* 2010 [97] | Yes | 1 |  | 1 | 1 | 1 | 1 | 1 | 6 | Low risk |
| Fletcher *et al.,* 2010 [117] | Yes | 1 |  | 1 | 1 | 1 | 1 | 1 | 6 | Low risk |
| Johnston *et al.,* 1997 [136] | Yes | 1 |  | 1 | 1 | 1 | 1 | 1 | 6 | Low risk |
| Kelly *et al.,* 2008 [100] | Yes | 1 |  | 1 | 1 | 1 | 1 | 1 | 6 | Low risk |
| Li *et al.,* 2019 [20] | Yes |  |  | 1 | 1 | 1 | 1 | 1 | 5 | Moderate risk |
| Luckin-Baldwin *et al.,* 2021 [135] | Yes | 1 |  | 1 | 1 | 1 | 1 | 1 | 6 | Low risk |
| Lum *et al.,* 2019 [105] | Yes | 1 |  | 1 | 1 | 1 | 1 | 1 | 6 | Low risk |
| Lum *et al.,* 2022 [112] | Yes | 1 |  | 1 | 1 | 1 | 1 | 1 | 6 | Low risk |
| Lundstrom *et al.,* 2017 [109] | Yes | 1 |  | 1 | 1 | 1 | 1 | 1 | 6 | Low risk |
| Mikkola *et al.,* 2007 [137] | Yes |  |  | 1 | 1 | 1 | 1 | 1 | 5 | Moderate risk |
| Mikkola *et al.,* 2011 [102] | Yes |  |  | 1 | 1 | 1 | 1 | 1 | 5 | Moderate risk |
| Millet *et al.,* 2002 [103] | Yes | 1 |  | 1 | 1 | 1 | 1 | 1 | 6 | Low risk |
| Paavolainen *et al.,* 1999 [8] | Yes |  |  | 1 |  | 1 | 1 | 1 | 4 | Moderate risk |
| Pellegrino *et al.,* 2016 [12] | Yes | 1 |  | 1 | 1 | 1 | 1 | 1 | 6 | Low risk |
| Piacentini *et al.,* 2013 [19] | Yes | 1 |  | 1 | 1 | 1 | 1 | 1 | 6 | Low risk |
| Saunders *et al.,* 2006 [113] | Yes | 1 |  | 1 | 1 | 1 | 1 | 1 | 6 | Low risk |
| Sedano *et al.,* 2013 [115] | Yes | 1 |  | 1 | 1 | 1 | 1 | 1 | 6 | Low risk |
| Skovgaard *et al.,* 2014 [104] | Yes | 1 |  | 1 | 1 | 1 | 1 | 1 | 6 | Low risk |
| Spurrs *et al.,* 2003 [107] | Yes | 1 |  | 1 | 1 | 1 | 1 | 1 | 6 | Low risk |
| Štohanzl *et al.,* 2018 [111] | Yes | 1 |  | 1 | 1 | 1 | 1 | 1 | 6 | Low risk |
| Støren *et al.,* 2008 [99] | Yes | 1 |  | 1 | 1 | 1 | 1 | 1 | 6 | Low risk |
| Trowell *et al.,* 2021 [119] | Yes | 1 | 1 | 1 | 1 | 1 | 1 | 1 | 7 | Low risk |
| Turner *et al.,* 2003 [106] | Yes | 1 |  | 1 | 1 | 1 | 1 | 1 | 6 | Low risk |
| Vikmoen *et al.,* 2016 [98] | Yes | 1 |  | 1 | 1 | 1 | 1 | 1 | 6 | Low risk |
| Vorup et al., 2016 [96] | Yes | 1 |  | 1 | 1 | 1 | 1 | 1 | 6 | Low risk |

N1: eligibility criteria were specified; N2: subjects were randomly allocated to groups; N3: allocation was concealed; N4: the groups were similar at baseline regarding the most important prognostic indicators; N5: measures of at least one key outcome were obtained from more than 85% of the subjects initially allocated to groups; N6: all subjects for whom outcome measures were available received the treatment or control condition as allocated or, where this was not the case, data for at least one key outcome was analysed by “intention to treat”; N7: the results of between-group statistical comparisons are reported for at least one key outcome; N8: the study provides both point measures and measures of variability for at least one key outcome; Risk of bias: ≥ 6 points = “low risk”, 4 to 5 points = “moderate risk”, and ≤ 3 points = “high risk”. Items 5 to 7 of the original scale were removed due to the infrequency of blinding of subjects, evaluators, and researchers in supervised exercise interventions.

**Electronic Supplementary Material Table S3**

**Article tittle:** Effect of strength training programs in middle- and long-distance runners’ economy at different running speeds: A systematic review with meta-analysis

**Author names:** Cristian Llanos-Lagos, Rodrigo Ramirez-Campillo, Jason Moran, Eduardo Saez de Villarreal

**Affiliation and e-mail of the corresponding author:**

MSc. Cristian Llanos-Lagos

Universidad Pablo de Olavide

Physical Performance Sports Research Center

41704 Sevilla

Spain

Email: cfllalag@alu.upo.es

ORCID: 0009-0003-6906-4977

**Table S3**. Results of meta-regression and subgroup analyses in search for possible moderators on combined methods training including on running economy

| **Combined methods training** | n groups | J | *β_0_* Hedges' *g* (SE) | CI 95% | *t_0_*(*df*), *p* value | *β_1_* Hedges' *g* (SE) | CI 95% | *t_1_*(*df*), *p* value | *F*(*df_1_, df_2_*), *p* value |
| --- | --- | --- | --- | --- | --- | --- | --- | --- | --- |
| Subject characteristics |  |  |  |  |  |  |  |  |  |
| Sex | 8 | 18 |  |  |  |  |  |  | *F*(2, 15) = 1.214, *p =* 0.325 |
| Male | 3 | 7 | -0.624 (0.269) | -1.198 - -0.051 | ***t*(15) = -2.321, *p =* 0.035** |  |  |  |  |
| Female | 1 | 2 | -0.906 (0.513) | -1.999 - 0.186 | *t*(15) = -1.768, *p* = 0.097 | -0.282 (0.579) | -1.516 - 0.952 | *t*(15) = -0.487, *p =* 0.633 |  |
| Male-Female | 4 | 9 | -0.207 (0.219) | -0.673 - 0.259 | *t*(15) = -0.946, *p =* 0.359 | 0.418 (0.347) | -0.321 - 1.156 | *t*(15) = 1.205, *p =* 0.247 |  |
| Age | 8 | 18 | -0.804 (0.607) | -2.090 - 0.483 | *t*(16) = -1.324, *p =* 0.204 | 0.015 (0.022) | -0.033 - 0.062 | *t*(16) = 0.648, *p =* 0.526 | *F*(1,16) = 0.420, *p =* 0.526 |
| Body mass | 8 | 18 | -1.517 (1.302) | -4.278 - 1.243 | *t*(16) = -1.165, *p =* 0.261 | 0.017 (0.020) | -0.025 - 0.058 | *t*(16) = 0.845, *p =* 0.411 | *F*(1,16) = 0.714, *p =* 0.411 |
| Height | 8 | 18 | 1.059 (5.635) | -10.886 - -0.008 | *t*(16) = -0.264, *p =* 0.795 | -0.008 (0.032) | -0.077 - 0.060 | *t*(16) = -0.264, *p =* 0.795 | *F*(1,16) = 0.070, *p =* 0.273 |
| VO_2_max | 8 | 18 | 1.037 (1.534) | -2.216 - 4.290 | *t*(16) = -0.959, *p =* 0.352 | -0.024 (0.026) | -0.079 - 0.030 | *t*(16) = -0.959, *p =* 0.352 | *F*(1,16) = 0.919, *p =* 0.364 |
| Performance level | 8 | 18 |  |  |  |  |  |  |  |
| Moderately trained | 1 | 2 | -0.023 (0.527) | -1.146 - 1.100 | *t*(15) = -0.043, *p =* 0.966 |  |  |  |  |
| Well trained | 2 | 3 | -0.582 (0.397) | -1.427 - 0.264 | *t*(15) = -1.466, *p =* 0.163 | -0.559 (0.660) | -1.965 - 0.847 | *t*(15) = -0.847, *p =* 0.410 |  |
| Highly trained | 5 | 13 | -0.451 (0.222) | -0.924 - 0.022 | *t*(15) = -2.032, *p =* 0.060 | -0.428 (0.572) | -1.647 - 0.790 | *t*(15) = -0.749, *p =* 0.465 |  |
| Strength training experience | 6 | 12 |  |  |  |  |  |  | *F*(1,10) = 0.666, *p =* 0.434 |
| No | 2 | 5 | -0.265 (0.304) | -0.942 - 0.411 | *t*(10) = -0.873, *p =* 0.403 |  |  |  |  |
| Yes | 4 | 7 | -0.692 (0.426) | -1.642 - 0.258 | *t*(10) = -1.624, *p =* 0.136 | -0.427 (0.523) | -1.593 - 0.739 | *t*(10) = -0.816, *p =* 0.434 |  |
| Strength training intervention |  |  |  |  |  |  |  |  |  |
| Weeks | 8 | 18 | -0.405 (0.446) | -1.351 - 0.541 | *t*(16) = -0.908, *p =* 0.377 | -0.002 (0.038) | -0.082 - 0.078 | *t*(16) = -0.053, *p =* 0.958 | *F*(1,16) = 0.003, *p =* 0.958 |
| Sessions per week | 8 | 18 | 0.221 (0.856) | -1.593 - 2.035 | *t*(16) = 0.258, *p =* 0.799 | -0.270 (0.349) | -1.009 - 0.470 | *t*(16) = -0.773, *p =* 0.451 | *F*(1,16) = 0.597, *p =* 0.451 |
| Total sessions | 8 | 18 | -0.186 (0.531) | -1.312 - 0.939 | *t*(16) = -0.351, *p =* 0.730 | -0.010 (0.020) | -0.052 - 0.033 | *t*(16) = -0.478, *p =* 0.639 | *F*(1,16) = 0.229, *p =* 0.639 |
| Exercise sequence | 8 | 18 |  |  |  |  |  |  | *F*(1,16) = 0.003, *p =* 0.956 |
| Traditional | 4 | 10 | -0.418 (0.247) | -0.941 - 0.106 | *t*(16) = -1.692, *p =* 0.110 |  |  |  |  |
| Complex | 4 | 8 | -0.438 (0.259) | -0.987 - 0.112 | *t*(16) = -1.689, *p =* 0.111 | -0.020 (0.358) | -0.779 - 0.739 | *t*(16) = -0.056, *p =* 0.956 |  |
| Speed assessed |  |  |  |  |  |  |  |  |  |
| Absolute speed (continuous) | 7 | 17 | 1.726 (1.071) | -0.556 - 4.008 | *t*(15) = 1.612, *p =* 0.128 | -0.171 (0.084) | -0.349 - 0.007 | *t*(15) = -2.045, *p =* 0.059 | *F*(1,15) = 4.180, *p =* 0.059 |
| Absolute speed (category) | 7 | 17 |  |  |  |  |  |  | *F*(1,15) = 3.444, *p =* 0.083 |
| ≤ 12.00 km/h | 5 | 7 | -0.193 (0.217) | -0.657 - 0.270 | *t*(15) = -0.889, *p =* 0.388 |  |  |  |  |
| > 12.00 km/h | 5 | 10 | -0.667 (0.208) | -1.111 - -0.222 | *t*(15) = -3.198, *p =* 0.006 | -0.473 (0.255) | -1.017 - 0.070 | *t*(15) = -1.856, *p =* 0.083 |  |
| U-shaped RE-speed relationship (category) | 7 | 17 |  |  |  |  |  |  | *F*(2,14) = 1.365, *p =* 0.287 |
| < 11.50 km/h | 2 | 2 | -0.068 (0.341) | -0.800 - 0.664 | *t*(14) = -0.200, *p =* 0.845 |  |  |  |  |
| 11.50 - 14.50 km/h | 7 | 14 | -0.457 (0.162) | -0.804 - -0.109 | ***t*(14) = -2.818, *p =* 0.014** | -0.388 (0.346) | -1.130 - 0.353 | *t*(14) = -1.123, *p =* 0.280 |  |
| > 14.50 km/h | 1 | 1 | -1.222 (0.652) | -2.621 - 0.176 | *t*(14) = 1.578, *p =* 0.137 | -1.154 (0.732) | -2.723 - 0.415 | *t*(15) = -1.578, *p =* 0.137 |  |
| Speed relative to AT | 4 | 8 |  |  |  |  |  |  | *F*(1,6) = 0.095, *p =* 0.768 |
| ≤ AT | 4 | 7 | -0.159 (0.167) | -0.567 - 0.248 | *t*(6) = -0.956, *p =* 0.376 |  |  |  |  |
| > AT | 1 | 1 | -0.159 (0.516) | -1.195 - 1.195 | *t*(6) = -0.001, *p =* 1.000 | 0.159 (0.516) | -1.103 - 1.422 | *t*(6) = 0.309, *p =* 0.768 |  |
| Speed relative to VO2max | 7 | 17 | 0.550 (1.216) | -2.042 - 3.142 | *t*(15) = 0.452, *p =* 0.658 | -0.013 (0.016) | -0.048 - 0.021 | *t*(15) = -0.832, *p =* 0.419 | *F*(1,15) = 0.692, *p =* 0.419 |

In the subgroup analysis (categorical variables), the first variable of the category was considered as the reference. AT, anaerobic threshold; n groups: number of experimental groups; SE, standard error; CI, confidence Interval; df, degree of freedom. Results in bold represent a significant effect (α = 0.05).

**Electronic Supplementary Material Figure S1**

**Article tittle:** Effect of strength training programs in middle- and long-distance runners’ economy at different running speeds: A systematic review with meta-analysis

**Author names:** Cristian Llanos-Lagos, Rodrigo Ramirez-Campillo, Jason Moran, Eduardo Saez de Villarreal

**Affiliation and e-mail of the corresponding author:**

MSc. Cristian Llanos-Lagos

Universidad Pablo de Olavide

Physical Performance Sports Research Center

41704 Sevilla

Spain

Email: cfllalag@alu.upo.es

ORCID: 0009-0003-6906-4977

**Fig S1.** Funnel plot of studies of different types of strength training that analysed running economy.


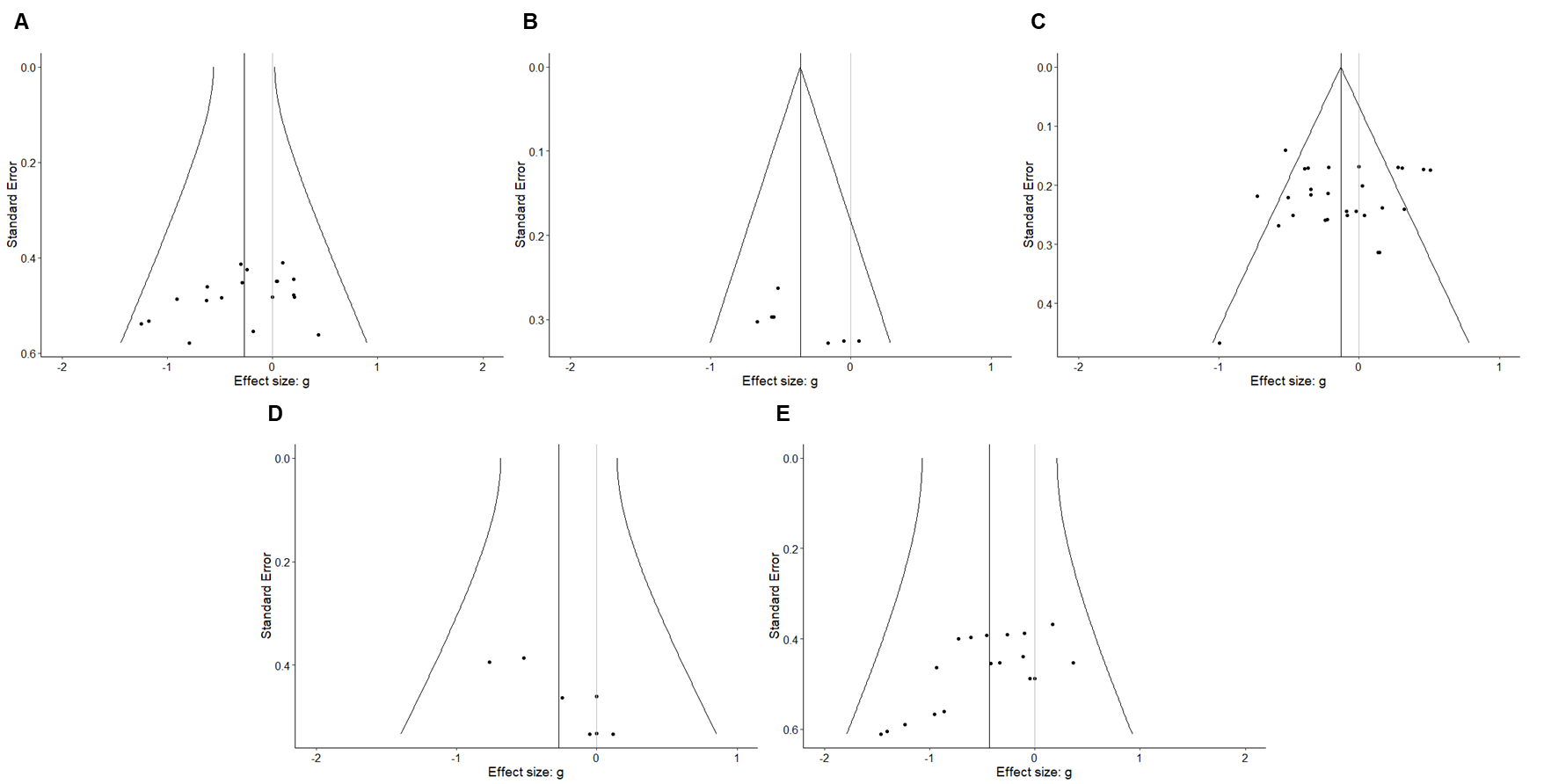


A: high load training; B: submaximal load training; C: plyometric training; D: isometric training; E: combined methods training.
